# Engineering Tunable, Low Latency Spatial Computation with Dual Input Quorum Sensing Promoters

**DOI:** 10.1021/acssynbio.4c00068

**Published:** 2024-05-23

**Authors:** Jure Tica, Haobin Chen, Shulei Luo, Manman Chen, Mark Isalan

**Affiliations:** † Department of Life Sciences, Imperial College London, London SW7 2AZ, U.K.; ‡ Imperial College Centre for Synthetic Biology, Imperial College London, London SW7 2AZ, U.K.

**Keywords:** spatial synthetic biology, genetic circuits, engineering intercellular signaling, mathematical modeling

## Abstract

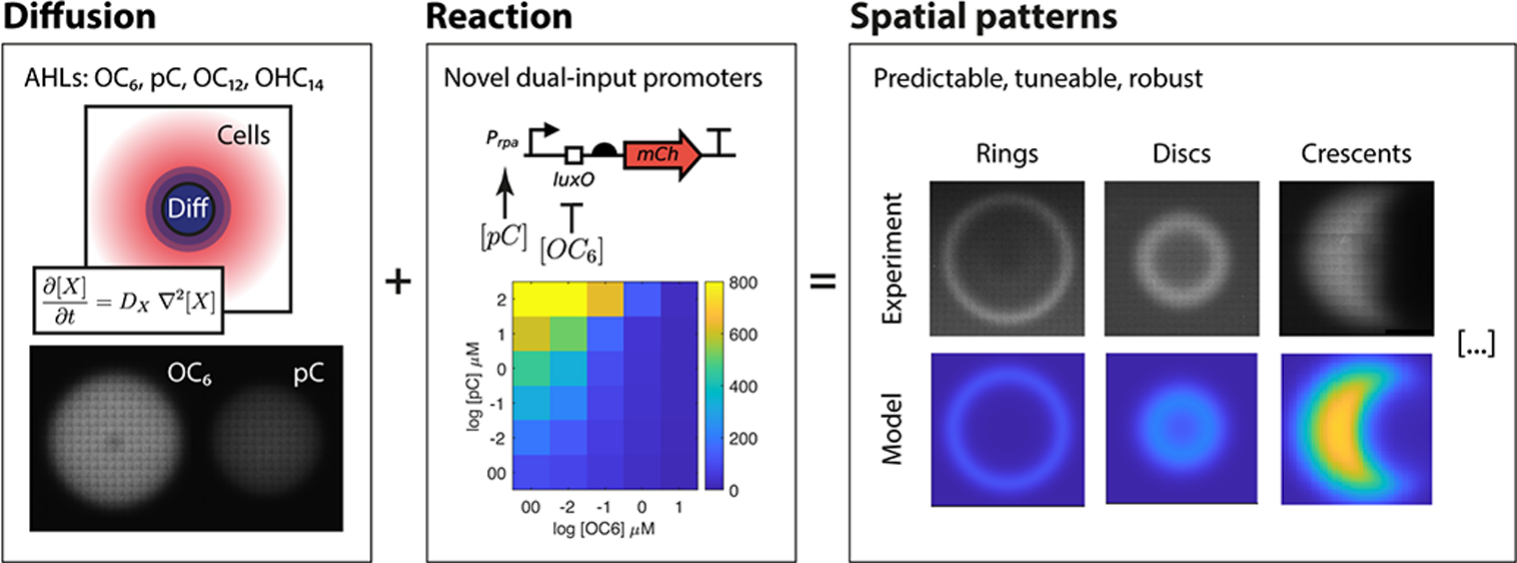

Quorum sensing signals have evolved for population-level signaling in bacterial communities and are versatile tools for engineering cell–cell signaling in synthetic biology projects. Here, we characterize the spatial diffusion of a palette of quorum sensing signals and find that their diffusion in agar can be predicted from their molecular weight with a simple power law. We also engineer novel dual- and multi-input promoters that respond to quorum-sensing diffusive signals for use in engineered genetic systems. We engineer a promoter scaffold that can be adapted for activation and repression by multiple diffusers simultaneously. Lastly, we combine the knowledge on diffusion dynamics with the novel genetic components to build a new generation of spatial, stripe-forming systems with a simplified design, improved robustness, tuneability, and response time.

## Introduction

The advent of novel synthetic genetic components, or “parts”,^1^ has been at the core of synthetic biology research. Parts development has continually enabled the engineering of increasingly more complex and refined circuitry, including logic gates,^2,3^ light-sensing systems,^4^ intercellular communication channels,^5,6^ spatial circuits,^7−10^ and beyond.

Because of their uses in controlling gene expression, much interest has been invested in the development of orthogonal inducible promoters for the simultaneous tuning of multiple genes with exogenous small molecules.^6^ These signal-sensing units were soon combined with synthesis pathways for the endogenous production of signaling factors.^5^ The combination of signal sensing and production endows cells with the capacity to regulate their communication with neighboring cells as a function of some inner computation. In this way, the components are particularly versatile as building blocks of engineered, spatially distributed gene expression systems^11^ and could enable the engineering of complex spatial patterns.

To expand this toolkit, this study aimed to build promoters that enable the integration of multiple small-molecule diffusive inputs. Multi-input promoters were previously engineered for intracellular transcription factors^2^ but not for diffusible inducers. This can reduce the need to introduce additional genes to perform the computations, which often complicates circuit implementation and increases metabolic burden.^10,13^ Additionally, it can make models more difficult to study by introducing additional equations and parameters.^7,9^

To demonstrate the advantages of promoters that integrate multiple diffusive inputs, we built simple stripe-forming systems using a single dual-input promoter expressing a reporter gene. In the past, the simplest stripe-forming systems were typically encoded with three-component networks, including diffuser-sensing modules and at least two transcription factors for signal integration.^7,10^ Here, we simplify these designs: our stripe-forming systems are implemented with a single multi-input promoter modulated by two diffusers.

After working with several types of communication systems – including salicylate, protocatechuate, and 3,4-dihydroxybenzoate^11,14^ – we find the family of acyl homoserine lactone (AHL) signals to be most versatile for use in *Escherichia coli*. Some of the disadvantages of nonquorum sensing systems include (1) insufficiently strong synthesis pathways that lead to poor activation of downstream promoters (salicylate, protocatechuate); (2) the need to express larger multigene synthesis pathways (3,4-dihydroxybenzoate, naringenin); and (3) unsuitable sensing components that suffer from high rates of background expression (3,4-dihydroxybenzoate).^5,6^ We also previously encountered problems related to poor inducibility (low fluorescent response) and strain- and context-dependence. By contrast, acyl homoserine lactones provided a set of reliable signals with a suitable range of different diffusion properties^11^ to enable useful diffuser-dependent multi-input promoter engineering.

First, however, we needed to develop a novel quantitative diffusion assay, with which we show that the signals act on different spatial scales and diffuse at different rates, dependent on their molecular weight.^12^ The systems also exhibit different types of promoter response functions and show different levels of specificity. With the calculated diffusion and kinetic parameters, we accurately predicted the behavior of the engineered stripe-forming system.

## Results

### Diffusion Characterization and the Lawn Assay

Various approaches have been developed to measure molecular diffusion in biofilms. Even though laborious, the use of chromatography and mass spectrometry was common in determining the sampled local concentrations of diffusing species, needed for the parameterization of diffusion models.^15^ The advent of FRAP and fluorescence microscopy simplified diffusion tracing.^16^ In synthetic biology, the diffusion of quorum sensing small-molecule signals was successfully measured in lawns of bacteria grown on agar plates, which were engineered to respond to the diffusers by expressing fluorescent reporter genes.^9^ However, a thorough assessment of the validity and working limits of these approaches has not been performed. This study evaluates these approaches and proposes a novel diffusion assay that circumvents some of their limitations.

**Figure 1:**
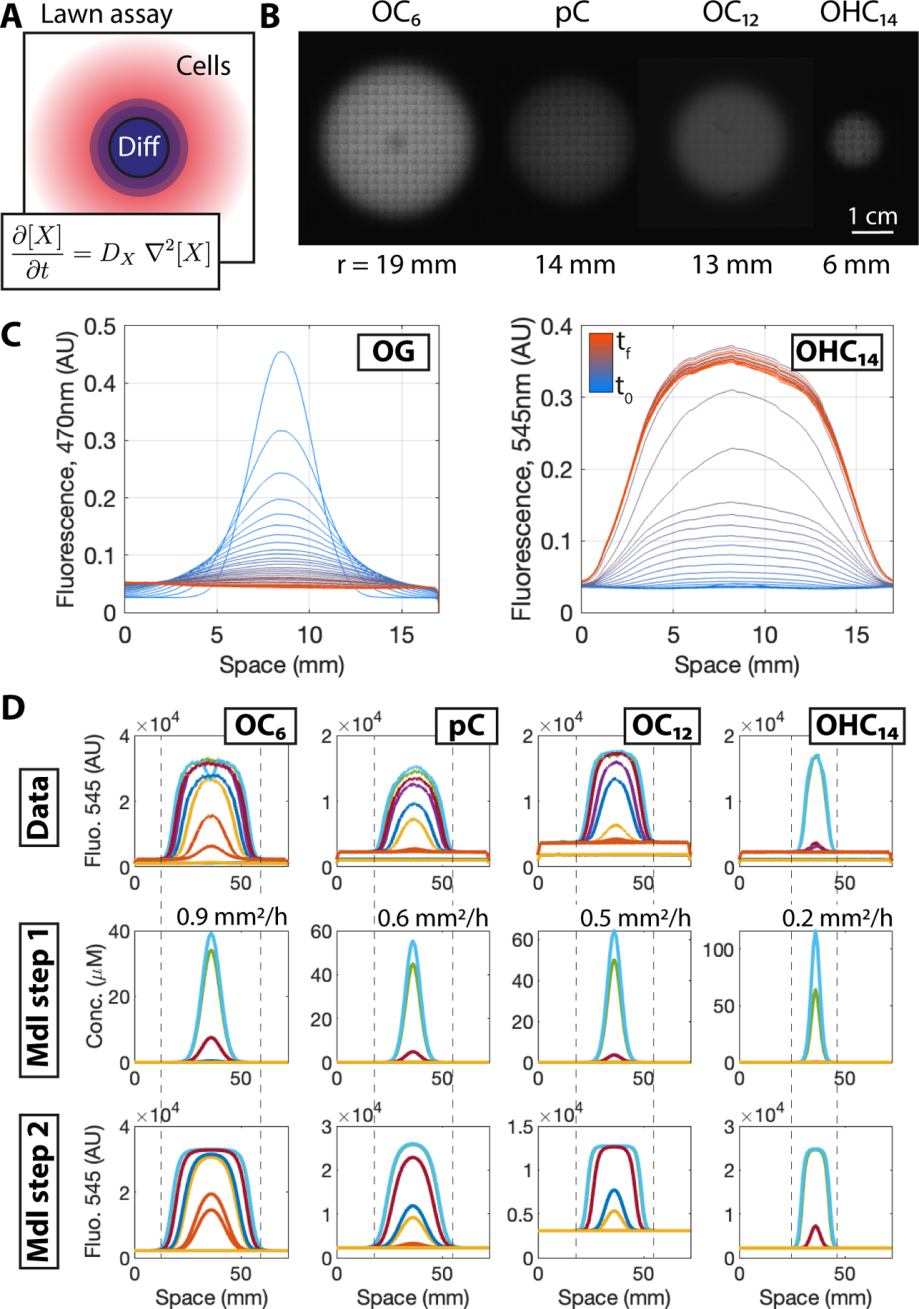
Lawn diffusion assay. (A) Schematic of the assay, where a lawn of cells responds to a diffuser by expressing a fluorescent reporter (mCherry). The reporter plasmid of the sensor strains consists of an mCherry gene linked downstream to appropriate promoters, sensing the corresponding signals. The rate of diffusion can be calculated from the spatial expression profile by using the equation shown. (B) Snapshots of lawn-based diffusion assays 10 h after diffuser administration. The radius (*r*) of the fluorescent area is shown in mm. (C) Fluorescence after the administration of a small droplet containing a mixture of OG488 (fluorescent dye; OG) and OHC14 (AHL) to the center of a lawn. The fluorescence of OG488 gradually disperses through a process of classical diffusion. The OHC14 signal also diffuses and induces the expression of mCherry from the lawn cells. Each curve is 30 min apart, where the initial time point is in blue (*t*_0_) and final one in red (*t*_f_). The plots were smoothed with a moving average filter to remove the tiling artifacts. (D) The first row (Data) shows cross sections of the fluorescent discs of (B). The second row (Mdl Step 1) shows the diffusion PDE simulations with the best-fit *D*_X_ values; these are the spatial concentration profiles of the diffusers. The third row (Mdl Step 2) shows the spatial fluorescence profiles obtained by feeding the curves of the second row (Mdl Step 1) through Hill functions fitted to promoter dose-response data.

A standard lawn-based diffusion assay involves measuring the diffusion over a lawn of cells engineered to express a fluorescent reporter in response to the diffuser. This assay was previously used to quantify the diffusion of C4 and OC12.^9^ Here, it was performed for OC6, pC, OC12, and OHC14, four homoserine lactones with increasing molecular weight (Figure 1). The small-molecule fluorescent dye Oregon Green 488 (OG488) was included in the diffuser droplets as an independent tracer of diffusion that does not rely on reporter gene expression. The lawn cells were transformed with plasmids, where the mCherry fluorescent reporter was expressed from promoters that are induced by the diffusers: Plux for OC6, Prpa for pC, Plas for OC12, and Pcin for OHC14. A droplet containing the diffuser at a concentration of 10^3^ μM was added to the center of the lawn, and the fluorescence response was measured over a period of 20 h with a microscope.

Approximately 10 h after inducer application, the four molecules produced discs of fluorescence of different sizes and intensities (Figure 1A). The fluorescent reporter gene and the OG488 tracer molecule showed different spatiotemporal dynamics (Figure 1B). The fluorescent tracer gradually dispersed away from the point of application and moved toward a spatially homogeneous state, as expected from a classical diffusion process. On the other hand, the reporter gene was initially absent throughout the spatial domain, gradually increased toward a bell-shaped spatial distribution, and ceased to change further. Even though the quorum-sensing inducer that triggers the fluorescence likely reached a homogeneous distribution throughout the spatial domain, following a similar diffusion trajectory to that of OG488, this is not reflected in the associated fluorescence response. This suggests that the lawn ceases to dynamically respond to the inducer after some time.^9^ Overall, the four molecules produce responses with differing spatiotemporal dynamics, which likely depend on the molecules’ diffusive properties and on the kinetics of the cellular response.

Next, a computational model was fitted to the experimental data to determine the diffusion rates of the four molecules (Figures 1C, S1, Table 1). The model involved a two-step approach. First, the diffusion partial differential eq (eq 1) was simulated in 2*D* space to determine the spatial concentration profiles of the diffusers (Figure 1C, Mdl Step 1). Second, these concentration profiles were transformed to fluorescence by feeding them through Hill functions (eq 2) that map the inducer concentration to fluorescence (Figure 1C, Mdl Step 2). While simulating the diffusion equation of the first step, the diffusion coefficient was adjusted so that the model fitted the experimental data.
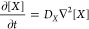
1

**Table 1:** Diffusion Parameters Derived from Experimental Data, Where ***D*** Is the Diffusion Coefficient, and ***r*** Is the Maximum Range of Diffusion^a^

	*D*_lawn_ (mm^2^/h)	*D*_dist_ (mm^2^/h)	*r*_lawn_ (mm)	*r*_dist_ (mm)
**OC6**	0.9	2.0	19	33
**pC**	0.6	1.2	14	23
**OC12**	0.5	1.2	13	21
**OHC14**	0.2	0.6	6	15

a The range of diffusion ***r*** was quantified by measuring the diameter of the fluorescent discs in the lawn assay and by taking the highest distance for which a fluorescent response was recorded in the distance assay (D values to 1 d.p.)

The Hill functions (eq 2) were calculated with dose–response data measured independently by inducing the cells with known concentrations of the inducer (Figure S2). A lawn of cells was prepared containing a spatially homogeneous, known concentration of the inducer, and fluorescence was measured after it reached steady state. These fluorescence measurements were fitted with eq 2, where α is the background expression in the absence of an inducer, *V*_*m*_ are the maximally regulated expression levels, *K*_*m*_ is the inducer concentration leading to a half-maximal response, and *n* is the Hill coefficient determining the slope of the curve. An induction in positive concentration has *n* > 0, whereas a repression has *n* < 0. The fits and parameters are presented in Figure S2 and Table S1.
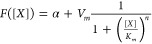
2

As explained earlier, the fluorescence of the lawn ceases to dynamically respond to the inducer over time (Figure 1B). The loss of responsivity was further tested by inducing the lawn with an increasing time delay after plating. Gradually decreasing fluorescence levels were observed with longer time delays between plating and induction (Figure S3). Eventually, when induction was delayed by approximately 10 h for OC6 and 16 h for OHC14, a fluorescent response was no longer detected. These results are consistent with the behavior of the lawns in response to a droplet of diffuser, where the fluorescence stopped changing after reaching a spatial bell-shaped distribution (Figure 1B). Hence, the fluorescent response is likely to be a snapshot of diffuser concentration at a particular time point, which is difficult to determine. In our simulations, we set this to 10 h, consistent with the dynamics of Plux’s response to OC6 (Figure S3). This decline in the response of the lawn is consistent with other studies, where the effect seems less pronounced, probably due to a lower temperature of 30 °C as opposed to 37 °C in our study.^9^ To measure diffusion more reliably, we developed a novel diffusion assay which only relies on the fluorescence response at the earliest time points, immediately after the diffuser reaches the cell, circumventing some of the problems related to the loss in responsivity.

### Diffusion Characterization and the Distance-Based Assay

Developed to address the limitations of the lawn assay, the distance-based diffusion assay involves placing a droplet of inducer at increasing distances from a circular patch of reporter cells placed on top of the agar that respond to the inducer with fluorescent reporter expression. The diffusion coefficient can be estimated by measuring the time taken for diffusion across a particular distance. The time for diffusion is measured between the time of droplet application and the time when the fluorescence response rises above an “induction threshold”, which is manually selected to be just above the background fluorescence level, and is similar in concept to the crossing threshold in standard qPCR. The assay only relies on the initial phases of the fluorescence response, immediately after the diffuser reaches the cells. The key differences of the distance assay compared to the lawn assay are that it only relies on the initial cellular response of freshly dividing cells at the edge of the cell patch and that it measures diffusion in empty agar that does not contain cells that could interfere with the diffusive process.

**Figure 2:**
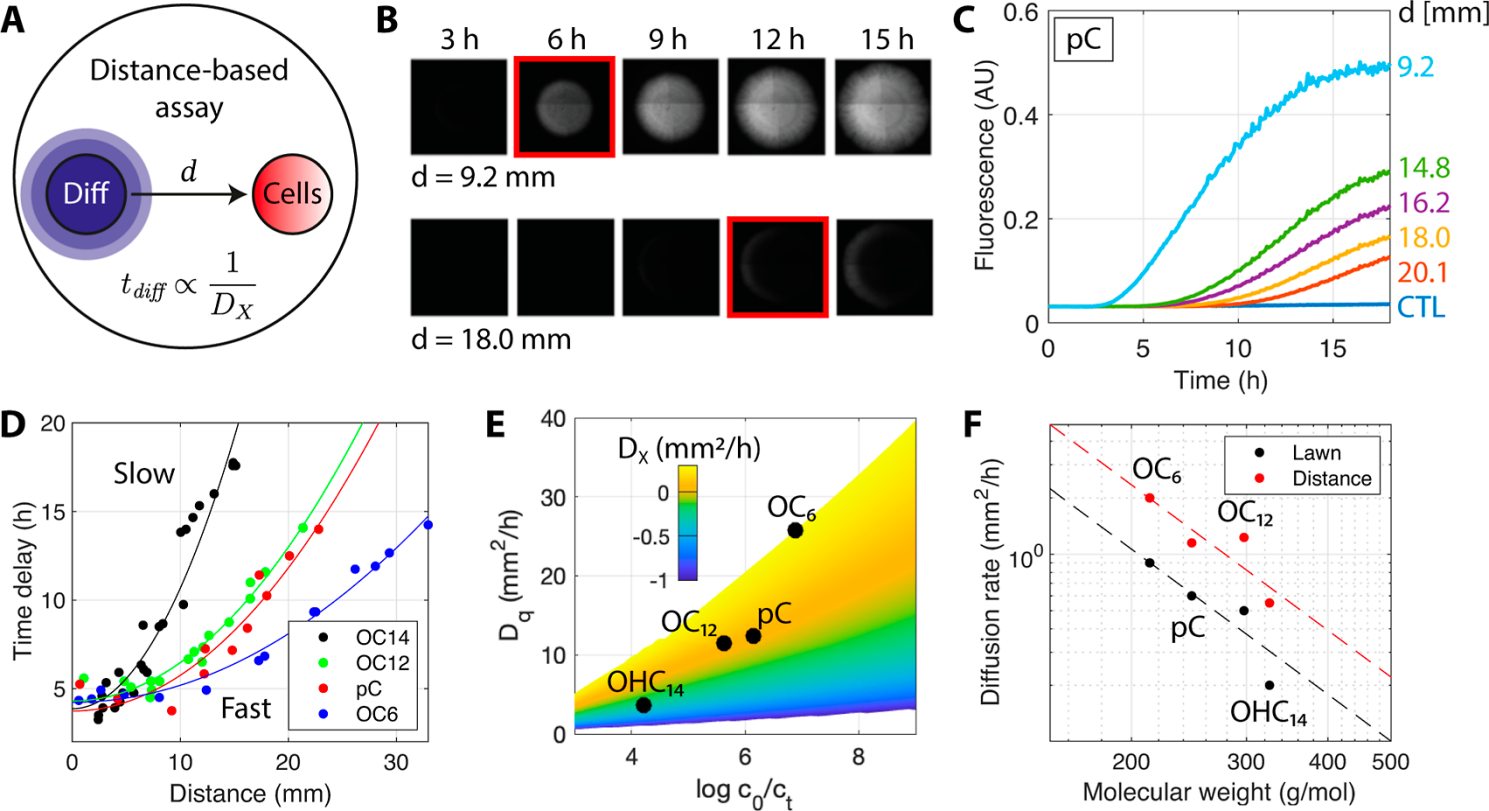
Distance-based assay. (A) Schematic of the assay, where a patch of cells responds to an inducer placed at a certain distance (*d*) by expressing the fluorescent reporter mCherry. The time delay for induction (*t*_diff_) is inversely proportional to the diffusion rate of the molecule (*D*_X_). (B) Fluorescence of cell patches after administering a small droplet of pC at a distance from the cells. The first time point where a response is recorded is highlighted in red. (C) Average fluorescence of the colonies in part B in time. The cells are induced with a gradually longer delay with an increasing distance from the diffuser droplet. The delay is given by the amount of time needed for diffusion to occur across this distance. With increasing distance, the fluorescence levels decrease because lower concentrations of the diffuser reach the cells. (D) Time delay for fluorescence induction increased with increasing distance between the cell patch and diffuser droplet. The rate of increase is determined by the diffusion rate *D*_X_. The data are fitted with the quadratic function in eq 4 and the *D*_q_ slope parameters are identified: *D*_q,OHC14_ = 3.7; *D*_q,OC12_ = 11.5; *D*_q,pC_ = 12.4; *D*_q,OC6_ = 25.8 mm^2^/h. The data are collected in at least three independent experiments for each diffuser, performed on different days. (E) The diffusion rate *D*_X_ is determined by using experimentally measured *D*_q_ and *c*_0_*/c*_t_ parameters. The calculated *c*_0_*/c*_t_ values for OHC14, OC12, pC and OC6 are 6.0 × 10^–2^, 2.3 × 10^–3^, 7.2 × 10^–4^, and 1.3 × 10^–4^ μM, respectively. Only diffusion rates *D*_X_ in the range of [0.1, 2] were simulated and plotted. (F) Diffusion rates are inversely proportional to the molecular weight. The relationship is described well with a power law.

The distance-based assay was performed for the following quorum-sensing signals: OC6, pC, OC12, and OHC14 with mCherry reporters regulated by Plux, Prpa, Plas, and Pcin, respectively (Figure 2). The fluorescence of the reporter colonies was measured with a fluorescence microscope every 5 min, up to 18 h after diffuser application. The average fluorescence levels of the colonies reveal that with increasing distance, the fluorescence crosses the induction threshold with an increasingly greater time delay (Figure 2BCD). Furthermore, the final fluorescence levels progressively decrease with increasing distance because a reduced amount of diffusers reaches the cells (Figure 2BC).

The time taken for the fluorescence curves to reach the induction threshold was measured. This threshold was set to be just above the uninduced background measurements. The time delay of the fluorescence response (*t*_fluo_) was then plotted against the initial distance between the inducer and reporter (*d*) (Figure 2D). This time delay is a sum of the time needed for diffusion (*t*_diff_) and the time needed for the cells to respond (*t*_cell_) to the diffuser (eq 3). The cellular response delay (*t*_cell_) was treated as a constant, consistent with the measurements across the effective diffuser concentration range (Figure S4). Of important note, the distances *d* need to be chosen so that *t*_diff_ is not significantly smaller than *t*_cell_ or close to zero.
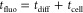
3

Next, a model was used to determine the diffusion rate (*D*_X_) from the relationships between *d* and *t*_diff_ for the four diffusers (Figure 2D). First, the diffusion PDE (eq 1) was simulated over a grid of *D*_X_ and *c*_0_*/c*_t_ parameters. The advancement of the threshold concentration that causes the minimal sensing event (*c*_t_) in reporter cells was plotted in time (Figures S5, S6). This relationship is equivalent to the curves in Figure 2D, and is consistent with eq 4, where ⟨*d*⟩^2^ is the mean square displacement of the inducer molecules, *D*_X_ is the diffusion rate, and *q*_*i*_ is a constant related to the dimensionality of the system, where *q*_*i*_ = 2, 4, 6 for 1-, 2- and 3-dimensional systems, respectively; we used *q*_*i*_ = 4.

Even though the data in Figure 2D is the displacement of the threshold concentration *c*_t_, rather than the mean square displacement, it is nonetheless consistent with the quadratic equation and is thus fitted with eq 4, where *D*_X_ is not the true diffusion rate, and is instead denoted as *D*_q_. *c*_0_ was the initial diffuser concentration in the applied droplets, and *c*_t_ was the inducer concentration that caused a minimal sensing event, determined as the inducer concentration that leads to a 5% promoter response in the dose–response assays (Figure S2). The relationship between *D*_q_, *c*_0_*/c*_t_, and *D*_X_, calculated using the PDE model over a grid of *c*_0_*/c*_t_ and *D*_X_ values, was used to estimate *D*_X_ for the four molecules, given the experimentally measured *D*_q_ and *c*_0_*/c*_t_ parameters (Figure 2E). The effective spatial spread of the fluorescence determined by *D*_q_ is positively correlated to *D*_X_ and *c*_0_*/c*_t_; this makes intuitive sense and means that the range of the fluorescence response can be increased both by increasing diffusion rate D_X_ or the sensitivity of the reporter promoter *c*_0_*/c*_t_. The diffusion coefficients are given in Table 1 and are consistent with those of the lawn assay.
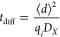
4

The diffusion rate of the molecules was inversely proportional to their molecular weight (Figure 2F). This molecular weight (*M*_w_) dependence was captured well with a power law (eq 5) by fitting the *k*_1_ and *k*_2_ parameters to the data. The diffusion rates calculated in both the distance and lawn assays were successfully fitted with a *k*_1_ of 9.0 × 10^5^ for the lawn assay, a *k*_1_ of 2.0 × 10^6^ for the distance assay, and a *k*_2_ of 2.6 for both (R^2^ = 0.90, F-stat. vs zero model *p* < 0.01). An equal *k*_2_ indicates that the *M*_w_ dependence is equivalent in empty agar and in cell suspensions. The *k*_1_ parameter was different in the two assays, owing to the diffusion rates in the distance assay being consistently higher than those in the lawn assay, by an average of 2.2-fold. This is also reflected by the larger range of diffusion (Table 1, *r*) in the distance assay and could be connected to the cell-dependent slowing of effective diffusion in lawns due to cellular uptake or other factors. The power law is a useful tool to estimate the diffusion rate of homoserine lactones in agar from their *M*_w_, in these particular experimental conditions at 37 °C. Caution should be exercised when extrapolating to other experimental conditions. For example, even changing the agar concentration was observed to affect diffusion rate (Figure S7).
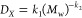
5

Next, we focused on developing novel dual-input promoters that can simultaneously be activated by more than one quorum sensing molecule, with which we could then build simple stripe-forming circuits. The diffusion coefficients calculated in this section are therefore used later to develop models of stripe formation.

### Novel Dual-Input, Diffuser-Regulated Promoters

The engineering of synthetic spatial systems relies on not only quantified diffusible components but also their respective regulatory genetic components, such as receptors and promoters. While numerous different diffusible signals have been engineered for synthetic biology, only simple regulatory promoters are available.^5,6,17^ Inspired by the multi-input promoters^2^ for the transcription factor λ cI, we engineered promoters that can be simultaneously activated and repressed by multiple quorum sensing molecules.^11,14^

**Figure 3:**
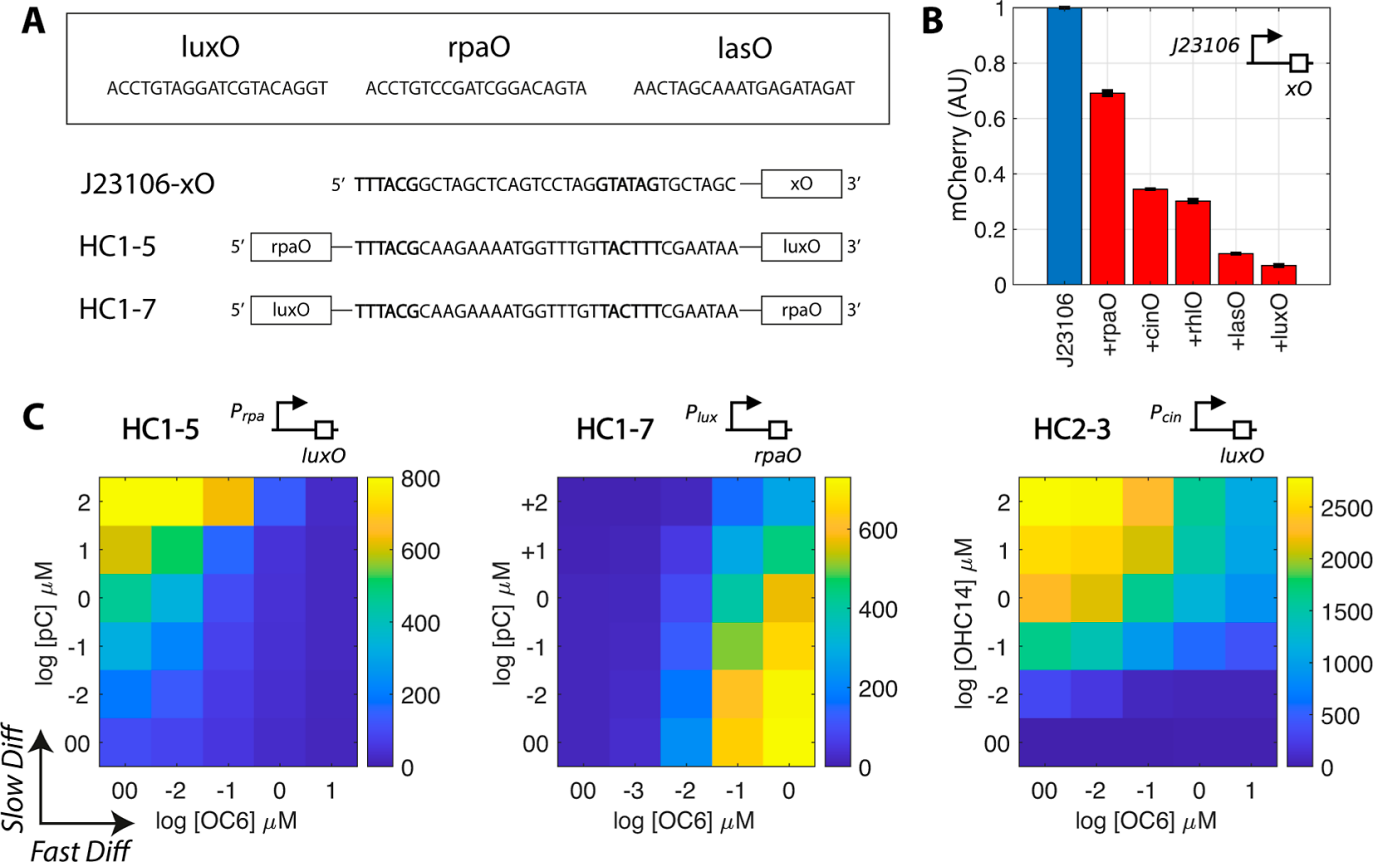
Repressible and dual-input quorum sensing promoters. (A) Five different quorum sensing receptor binding sites (rhlO, luxO, rpaO, lasO, and cinO) were introduced into existing promoter scaffolds to create quorum repressible promoters J23106-xO or dual-input promoters HC1–5 and HC1–7. (B) Repressible J23106-xO promoters were tested in liquid culture upon the addition of their respective repressors. Expression levels are relative to the uninhibited controls, which are assigned a value of 1. Inducer concentrations are 10 μM for OC6, pC, and OHC14, and 100 μM for C4 and OC12. The rpaO construct displays the weakest repression, whereas the lasO and luxO constructs display strong repression. (C) Dual input promoters HC1–5, HC1–7, and HC2–3. Promoter HC1–5 is induced by pC and completely repressed by OC6, with little background activity in the fully repressed state. Promoter HC1–7 is activated by OC6 and repressed by pC; there is some background activity in its fully repressed state. Promoter HC2–3 is strongly activated by OHC14 and repressed by OC6, where there is some background activity in the fully repressed state. Both axes are logarithmic, and the no-inducer controls are denoted by 00. All data measurements are taken at the 18 h time point; *n* = 3, mean ± SEM.

The first step was to engineer simple, repressible quorum sensing promoters. A recent study created OC6-repressible systems by placing a LuxR binding domain (luxO) into the core region of a constitutive promoter, between its −10 and −35 boxes.^17^ Here, we instead placed the receptor binding domains immediately downstream a constitutive J23106 promoter^1^ (Figure 3A). In this way, we designed and tested many different promoter variants (Figures S8–S11) and identified five repressible promoters, regulated by C4/rhlO, OC6/luxO, pC/rpaO, OC12/lasO, and OHC14/cinO through their respective receptors LuxR, RpaR, LasR, and CinR (Figure 3B).

The cinO that was used originally^6^ showed no activity and required optimization. We therefore fragmented the Pcin promoter and placed the short segments downstream of the constitutive J23106 promoter (Figure S9). Three of the tested fragments showed repression upon OHC14 addition. One of these three variants showed greatly reduced constitutive expression levels and was not considered (Figure S9, v3). The v1 variant featured the tightest repression in fold change by OHC14-CinR while maintaining a high constitutive expression level and was hence our preferred variant, also shown in Figure 3B.

Overall, repressible systems for C4/RhlR, OC6/LuxR, pC/RpaR, OC12/LasR, and OHC14/CinR were engineered and displayed varying levels of repression by their respective inducers (Figure 3B). The rpaO was least efficient at repressing the constitutive activity of the promoter, whereas the luxO was most efficient. The efficiency of repression likely relates to the binding affinity of the transcription factor to the operator site and to its efficacy at halting the progression of RNA polymerase.^18^

A quorum sensing operator site activates when placed upstream and represses when either placed into the core region or downstream a DNA sequence containing −10 and −35 regulatory regions.^5,17,19^ We used this to engineer dual-input promoters, where one quorum sensing input activates, whereas another represses.

After testing various designs, we initially obtained two functional dual-input promoters: HC1–5 and HC1–7 (Figures 3A, S10). By testing the promoters over a grid of inducer and repressor concentrations, we show that the HC1–5 promoter is activated by pC and repressed by OC6, whereas the HC1–7 promoter is activated by OC6 and repressed by pC (Figure 3C). A third dual-input promoter HC2–3 was engineered by placing the LuxR binding site immediately downstream the Pcin promoter (Figure S11). This yielded a promoter that is activated by OHC14 and repressed by OC6 (Figure 3C). The performance of all the tested variants is measured and summarized in Figures S10–S11. In our designs, promoters that are simultaneously strongly activated by the inducer and tightly repressed by the repressor were rare. Future research should focus on engineering strong, orthogonal operator sites and developing a toolbox of modular components.

These novel dual-input components can be applied to the engineering of simpler, more robust spatial systems with a greater ability to encode complexity. We next ventured to explore one such example through the engineering of a stripe forming system.

### Simplifying the Engineering of Stripe-forming Networks

The novel regulatory components are promising new tools that could markedly simplify the engineering of robust synthetic spatial systems. For example, in the past, stripe forming systems were engineered with three-node incoherent feedforward networks, implemented with an intracellular circuit of interacting transcription factors.^7,10^ The engineering of these systems can be laborious, as it requires the careful balancing of the various genetic components. In addition, they lack robustness as they are easily disturbed by metabolic burden caused by the simultaneous expression of multiple synthetic genes.^13^

An incoherent feed-forward system can be engineered by simply using a single dual-input promoter expressing a fluorescent reporter gene. No other intracellular circuitry is needed: the incoherent feed-forward motif is implemented with the two diffusers rather than by an intracellular genetic circuit. In this case, the system can be tuned by adjusting the relative concentrations of the activating and inhibiting diffusers in the applied droplet rather than by engineering circuit variants on the DNA level. Overall, this simplifies the engineering and tuning of the system, improves its response time, and leads to more robust and reproducible experiments.

The engineered system is based on the HC1–5 promoter of Figure 3C, which was chosen because it had the best dynamic range compared to the other two promoters HC1–7 and HC2–3 due to a strong level of activation by the inducer pC and tight repression by the inhibitor OC6.

**Figure 4:**
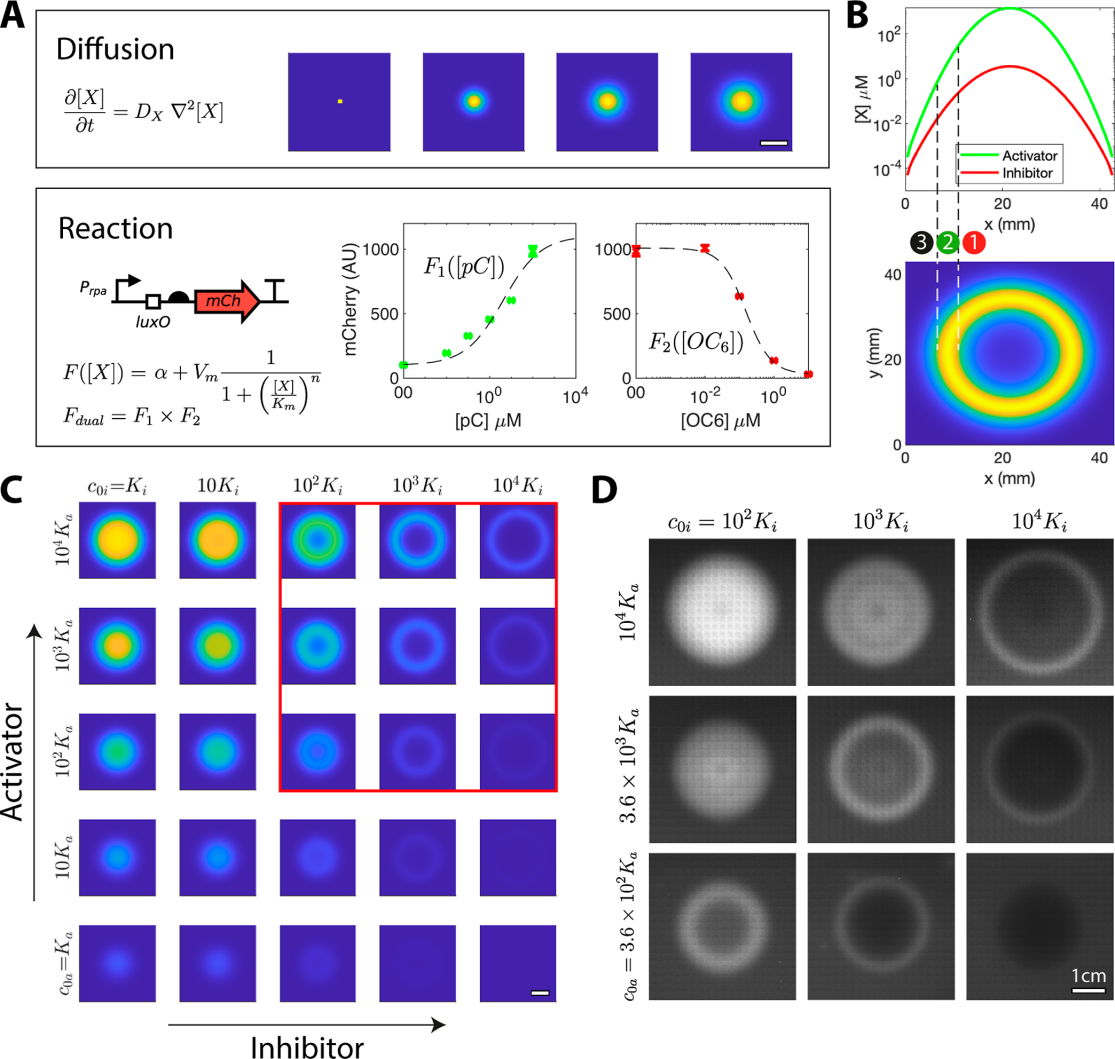
Stripe formation in an incoherent feedforward system with two diffusers. (A) Signals diffuse according to a simple diffusion process, modeled with a PDE over a 2D spatial domain. The signals act on a dual input promoter, where pC activates and OC_6_ represses, modeled with a product of Hill functions, where *F*_1_ is the activation function (*n >* 0), and *F*_2_ is the repression function (*n <* 0). *F*_1_ is parameterized with pC induction data in the absence of OC6 (green plot). *F*_2_ is parameterized to OC6 repression data (red plot) in the presence of near-maximal activation (10^2^ μM pC). These parameters were used to obtain the ring simulations in part (C). Mean ± SEM, *n* = 2. (B) The upper plot shows the diffusion of pC and OC_6_ from a small droplet containing both diffusers placed at the center of the spatial domain (*x*). The lower plot shows a ring of fluorescence expression obtained by feeding the concentrations of pC and OC6 at each spatial point into the dual input Hill function *F*_dual_. In the central region (1), inhibition wins; in the ring region (2) activation wins; in the surrounding region (3), there is no activation or inhibition. (C) The spatial fluorescence expression profile is tuned by changing the initial diffuser concentration: *c*_0a_ for the activator; *c*_0i_ for the inhibitor; *K*_a_ is *K*_m_ of the activator, whereas *K*_i_ is that of the inhibitor. (D) Rings were observed after applying a mixture of the activator and the inhibitor at the center of a homogeneous agar suspension of reporter cells containing HC1–5. The experiment is consistent with the model in (C), where the 9 experimental conditions are highlighted with a red square. The images were taken 24 h after induction. All scale bars are 1 cm. All model and microscope images have the same scale.

When cells containing the HC1–5 reporter were grown suspended in an agar lawn (Figure 4A), a ring of fluorescence expression formed in response to a small droplet of diffuser, containing a mixture of the activator pC and inhibitor OC6, applied to the center of the spatial domain (Figures 4B, S12). At small distances from the initial droplet, repression dominated, whereas at intermediate distances activation won out, resulting in a ring of fluorescence. At large distances, both diffusers dispersed, and there was again no activity in the system. This behavior was achieved only with a subset of activator/inhibitor initial concentration combinations, with the right balance between activation and repression (Figure 4CD). Tipping the system toward activation by increasing the activator at the expense of the inhibitor produced discs of fluorescence due to an absence of the central repressed region. On the other hand, tipping the system toward inhibition produced a circular shadow of repression in the center, with below-background expression levels. Overall, the behavior was consistent with our expectations and with the logic of the system.

A simple model was developed to explore the effects of changing the initial diffuser concentration. The model consisted of a diffusion and a reaction component (Figure 4A). First, the diffusers were modeled to undergo simple diffusion with a partial differential equation in 2*D* space using the diffusion rates derived in the lawn assay. Second, the activation levels of the multi-input promoter were calculated along the space grid using a dual-input Hill function. An activating Hill function *F*_1_ (eq 2, *n >* 0) was fitted to pC induction data in the absence of OC6 repression (Figures 4A, S13). An inhibiting Hill function *F*_2_ (eq 2, *n <* 0) was fitted to the OC6 repression data of the maximally activated pC response (Figures 4A, S13). The scaled product of the two Hill functions captured the HC1–5 response function well.

The model showed different behaviors depending on the concentration of the diffusers in the applied droplet, including small spots, larger circular discs, and rings of different sizes (Figure 4C). The model closely matched the experimental data (Figure 4D). Rings occurred with a specific combination of initial activator and inhibitor concentrations; ring diameter increased with increasing activator concentration, rings dispersed when activator concentration was not sufficiently high, ring visibility decreased with increasing inhibitor concentration, and circular discs occurred when initial activator concentration was high and inhibitor concentration was low. Overall, the system robustly and reproducibly generated rings and discs of fluorescence expression, which were closely captured by a reaction-diffusion model.

**Figure 5:**
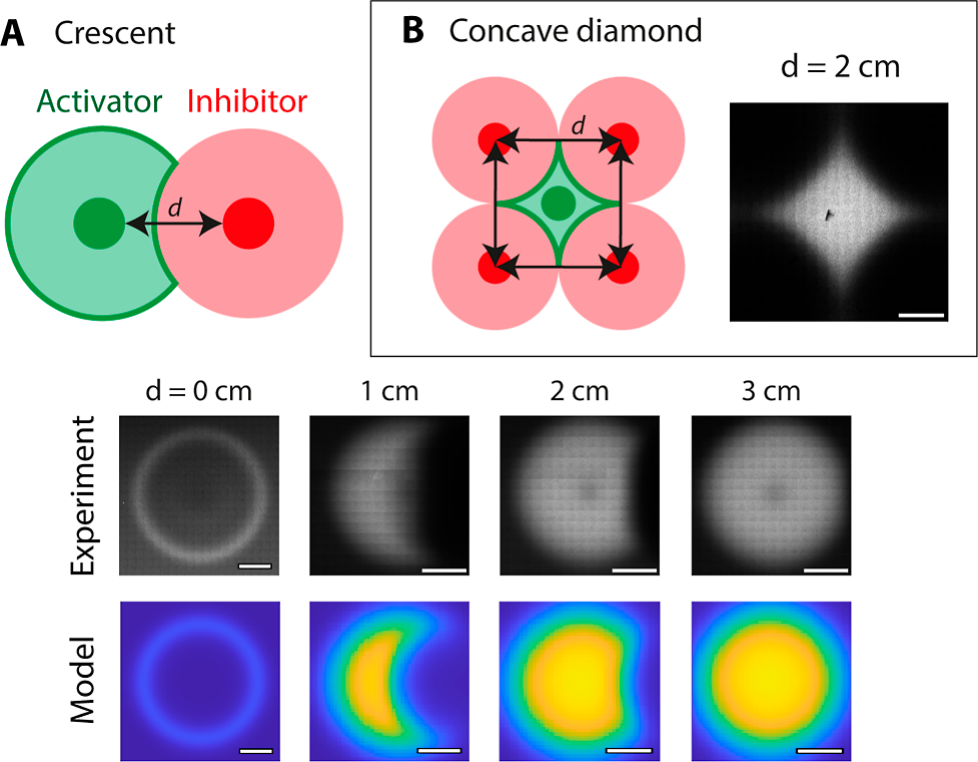
Crescents and concave diamonds. (A) Crescents are obtained when the distance between the activator (green) and inhibitor (red) droplets is increased. The shape of the crescent can be tuned by adjusting the distance between the two droplets. At a distance of 0 cm, a ring is obtained, as shown in Figure 4. Increasing this distance to 1 cm produces a half crescent, whereas increasing it further reduces the area of the inhibited region. Inhibition is absent at distances equal to or greater than 3 cm. (B) Concave diamonds are obtained by plating the inhibitor droplets in a square, where the activator droplet is in the middle. This creates four zones of repression at the four edges of the activated region, forming a concave diamond shape. The images were taken 24 h after induction. Scale bars are 1 cm.

Finally, crescents and concave diamonds were engineered by plating the activator and inhibitor droplets in different ways over a lawn of cells containing the HC1–5 reporter (Figure 5). Diffuser concentrations for both crescents and diamonds were 15 mM pC and 1.5 mM OC6. Crescents were obtained by plating the pC activator and OC6 inhibitor droplets at increasing distances (Figure 5A). At a 1 cm distance, the activated region was obscured by the inhibitor through the middle. At a distance of 2 cm, the inhibited region was smaller, whereas no inhibition was observed at 3 cm. Hence, the shape of the crescent can be tuned by plating the activator and inhibitor at a distance between 0 and 3 cm at the given concentrations. Concave diamonds were observed when four droplets of the inhibitor were plated on the edges of a square, with the activator droplet in the middle (Figure 5B). This occurred when the distance between the centers of the inhibitor droplets was 2 cm. Overall, various spatial patterns can be obtained by changing the initial condition.

The lack of elaborate genetic circuitry makes such systems much easier to implement, much more robust to extrinsic factors, less prone to metabolic burden, more tunable through exogenously regulated stimuli, and with an improved response time.

## Discussion

From the perspective of spatial synthetic biology,^7−10^ incorporating quantitatively studied diffusive components into synthetic circuits will unlock the possibility of engineering more sophisticated dynamics of patterning. The analysis and prediction of a complex patterning behavior usually involves the modeling of the circuit dynamics, where the quantitative measurement of the diffusion can parameterize the model and facilitate the process.^9^ As a representative example of a complex diffusion-mediated circuit, Turing patterning^20^ is based on the interaction between at least 2 diffusers in reaction-diffusion systems.^21,22^ Recently, a three-node Turing patterning circuit was engineered to form periodic spatial patterns, along with a quantitative model to analyze the circuit behavior.^23^ Understanding and reproducing such patterning processes from first principles can provide insights into regenerative medicine, developmental biology, and biomaterials.^24,25^

In the present research, we first examined a lawn-based assay to quantitatively measure diffusion on agar and then proposed a novel distance-based assay, which has several advantages. Both methods are based on fluorescence microscopy, which should be readily accessible to most laboratories. The assays were performed on a range of AHL molecules that are commonly used in synthetic biology: OC6, pC, OC12, and OHC14. We determined that OHC14 diffuses the slowest, whereas OC6 diffuses the fastest among the four molecules; this is to be expected given the relative sizes of the molecules, with OHC14 being the largest and OC6 the smallest.

An inversely proportional power law (eq 5) was found between the molecular weight and the fitted diffusion rates of the quorum sensing molecules, parameterized by an exponent of ∼2.6. While the exponent for an idealized spherical molecule is expected to be 1/3, departures from this value are common, where studies often report coefficients higher than 1/3 and as high as 2.^12,26,27^ Some of the reasons for this are that small molecules are often not spherical, may adopt a range of conformations, and significant interactions with the solvent are not uncommon.^28^ Rather than identifying a universal law for diffusion in biofilms, the power law derived here is proposed as a useful tool to approximate the diffusion rate from the molecular weight in a particular experimental setup for a particular class of molecules. Caution should be exercised when extrapolating our parameters to other experimental contexts or classes of molecules.

The distance-based assay exhibits several advantages: first, it conceptually captures the earliest, minimal sensing event, immediately after the first molecules of diffuser reach the reporter cells. Second, the sensing always occurs at the edge of the cell patch, where there are freshly dividing cells that can be relied upon to measure the response to a small amount of diffuser. Hence, the cell patch preserves the ability to respond to the stimuli throughout the experiment. On the other hand, a background signal was consistently observed for the reporter cells, which might be due to a combination of a noisy gene expression system and the background accumulation of the reporter.^13^ A manually set threshold was therefore introduced to exclude the noise range and to reliably identify the moment when induction occurs. To remove bias and pursue a more sensitive identification of induction, a noninducer control should be included in each experiment to subtract the background signal from the experimental data. This is particularly important for the longer distances, where induction can be low and is more difficult to identify from the data.

Three novel dual-input promoters, HC1–5, HC1–7, and HC2–3, were engineered using the previously characterized diffuser signals. They enable the integration of activating and repressing diffuser signals using a single promoter. For example, the HC1–5 promoter is activated by pC and repressed by OC6, whereas the HC1–7 promoter is activated by OC6 and repressed by pC. Such parts may be useful for synthetic biology projects such as engineering Turing patterns,^9,23^ where a single integrating promoter is much closer to the mathematical description of Turing.^21,22^ This is useful compared to the current methods of adding extra steps, such as inverters based on repressive transcription factors acting on downstream promoters, to get the dual activation-repression signaling required.^7,9,10^

The potential of the components to build simplified and robust systems was then demonstrated by a one-node system for stripe formation. Our system produces a “ring” stripe with a programmable radius and thickness simply by altering the concentration of diffusers in the initial combination being applied to the center, which can be straightforwardly simulated with the parameterized model. Compared to a three-node feedforward system with a similar function,^7,10^ our simpler system allows a more versatile programming of the pattern without incorporating any extra genetic components. The system was also shown to produce crescents by applying the initial droplets of two diffusers separately across a small distance, thereby creating a shifted inhibited area that “eclipse” the activated area. Applying the inhibiting diffuser at the four corners of the lawn with the activator in the center created a concave diamond. This illustrates the system’s versatility in producing a diverse range of patterns. In the future, circuits forming periodic patterns could also theoretically be engineered by introducing synthases of the diffusers involved into the genetic cascade.

One major advantage of the simplified one-node system is a reduction in response time. Each step in the transcription factor cascade of larger systems can create delays on a scale of minutes to hours^29,30^ and impact the robustness of the dynamics.^31,32^ In our system, computation occurs on the promoter level through the transcription factor and RNA polymerase binding events, which occur at a much faster time scale compared to transcription factor synthesis.^29^ In the future, computation could also be engineered through the competition of the diffusers at the receptor level. The integration between receptor-level regulation and promoter-level regulation could be used to engineer complex systems.

In conclusion, the dual-input promoters for quantified diffusing signals and our illustration of a one-node stripe-forming system can simplify downstream circuit designs for pattern formation. Furthermore, they can allow for a more accurate implementation of the design principles for classical reaction-diffusion systems. A simplified scaffold also allows a potentially higher level of integrated complexity and circuit function, which is advantageous in terms of biological component availability and avoiding metabolic burden. Finally, the study expands the toolkit of available genetic synthetic components and paves the way toward engineering simpler and more robust synthetic circuitry.

## Methods

### Cells and Media

*E. coli* 5α (NEB C2987) were used for cloning. MK01 cells^33^ were used in experiments. The primary outgrowth medium for fluorescence assays and microscopy was 2xYT (Sigma-Aldrich Y1003), LB (Sigma L2897) was used to make agar plates for cloning, and SOC (NEB B9020) was used for cell recovery after transformation.

### Electroporation of *E. Coli*

To prepare competent cells, cells were grown up in LB media, washed with ddH_2_O, resuspended in 10% glycerol in ddH_2_O, and centrifuged at 4000*g* at each step.^34^ Cells were electroporated with a pulse of 1.8 kV, 200 Ω, 50 μF, recovered in 500 μL SOC broth, incubated for 1 h at 37 °C, shaking, plated on LB agar with the appropriate antibiotics, and incubated overnight at 37 °C. To maximize transformation efficiency, the cells were first electroporated with the pCC1 receptor array plasmids, made electrocompetent using this protocol, and then transformed with the other plasmids.

Briefly, all the strains were transformed with (1) a pET plasmid containing the mCherry reporter gene expressed under the relevant promoter, whose sequences are listed in Tables S5–S7; (2) a pCC1R plasmid containing constitutively expressed receptor genes rpaR and cinR; (3) optionally a p15A plasmid harboring a constitutive luxR, lasR, or rhlR when needed.

### Molecular Cloning

DNA was amplified with Q5 Hot-Start Polymerase (NEB M0494), template DNA was removed with DpnI at 37 °C for 1 h, the reactions were run on a 1% (w/V) agarose gel in TAE buffer at 90 V for 1 h, and the bands were then extracted with the Monarch DNA Gel Extraction Kit (NEB T1020). The KLD kit (NEB M0554) was used for end-to-end ligations of a single fragment, whereas the HiFi assembly kit was used to assemble two or more fragments. The assembled constructs were then transformed into NEB 5α chemically competent cells, incubated overnight at 37 °C. On the following day, the colonies were analyzed with colony PCR. The colonies with the correct assemblies were grown overnight in 8 mL of 2xYT (16 mL for very low-copy pCC1 plasmids). The DNA was extracted with a Qiagen QIAspin MiniPrep kit (Qiagen 27,106). The volume of kit P1, P2, and N buffers was doubled for every 8 mL of bacterial culture. For pCC1 plasmids, two columns were used for each 16 mL sample. The plasmids were sequenced with the Eurofins SupremeRun Sanger service. The plasmids were deposited on Addgene (https://www.addgene.org/Mark_Isalan).

### Lawn-Based Diffusion Assay

MK01 cells electroporated with the desired plasmids were grown with orbital shaking at 37 °C to midexponential phase (OD_600_ 0.4–0.6). The cells were mixed 1:10 with dissolved 2xYT agar (1.4% w/V) and transferred to a single-well plate (Greiner 670190) using 20 mL of the cell-agar mixture per well. The inducers (10^3^ μM) were supplemented with 0.1 mM Oregon Green 488 (Cayman Chemical 35372, OG488). Once the agar solidified, an inducer droplet of 0.5 μL was applied at the center of each well. The mCherry and OG488 signals were imaged over a fixed region of the cell lawn at 30 min time intervals over 18 h with a Zeiss Axio Observer Z1 microscope. Imaging was carried out by using the built-in mCherry (610 nm) and OG488 (488 nm) channels. Settings for mCherry: light source HXP 120 V lamp, intensity 58%, exposure time 40 ms (150 ms for Plas/OC12 assays), exposure intensity 30%. Settings for OG488: light source HXP 120 V lamp, intensity 58%, exposure time 30 ms, and exposure intensity 30%. Each plate was incubated at 37 °C for up to 18 h.

The data were analyzed in MATLAB R2022a. The image data was first treated with a moving average filter, see Figure S1 for further information. This eliminated tiling artifacts and considerably reduced the noise. Next, Hill functions were fitted to the spatially homogeneous induction data to determine the relationship between inducer concentration and fluorescence, see Figure S2 for further details. A spatial PDE model was simulated with a Crank-Nicolson solver in 2*D* space with dimensions of 72 × 48 mm, equivalent to the size of the imaged region of the single-well plate used for the assay, with a 1 mm spacing between the adjacent points. The initial diffuser concentration (*c*_0_) was set to 10^3^ μM. The initial diffuser droplet was placed in the center of the spatial domain, with an experimentally measured radius of 1 mm. Flux was allowed across the boundaries, consistent with the experiment, where the size of the cell culture dish (128 × 86 mm) exceeds the size of the imaged and simulated area (72 × 48 mm). The time step was 0.01 h. Matrix LU decomposition was used to speed up computation, performed with the in-built MATLAB algorithm *lu*. The model produced spatial concentration profiles of the diffusers, which were converted to fluorescence by using the Hill functions fitted in the previous step. The diffusion rate *D*_X_ of the diffusion PDE was adjusted to fit the experimental data for OC6, pC, OC12, and OHC14.

### Distance-Based Diffusion Assay

A single colony of MK01 cells, electroporated with the desired plasmids, was picked and resuspended in 20 μL of 2xYT to make a cell suspension. The inducer was mixed 1:10 with 10 mg/mL trypan blue (Sigma T6146) to a final concentration of 10^3^ μM to create the inducer droplet. Cell droplets of 0.5 μL and inducer droplets of 0.5 μL were applied at increasing distances on individual wells of a six-well agar plate (Greiner 657160). Each well contained 2 mL of 2xYT agar (VMR 84609, 1.4% w/V). A one-well plate (Greiner 670190) was also used to test longer distances between the droplets in OC6 assays (>25 mm). The mCherry signal was imaged over a fixed region of each cell patch at 5 min time intervals over 18 h with a Zeiss Axio Observer Z1 microscope. Imaging was carried out using the built-in mCherry channel (610 nm), light source HXP 120 V lamp, intensity 58%, exposure time 40 ms (150 ms for Plas/OC12 assays), and exposure intensity 30%. The plate was incubated at 37 °C for up to 18 h.

The data were analyzed in MATLAB R2022a. The intensity of the signal was quantified by the average pixel intensity of the cell patch images. The dark, nonfluorescent regions of the images were excluded from the averaging by computing a binary mask of the images with the built-in MATLAB algorithm *imbinarize*. This was especially important for weakly induced patches, containing larger dark areas that could potentially skew the averages. The initial distances between the inducer droplet and the cell patch (*d*) were measured in Fiji from their bright-field images taken at the initial time point. A manually set threshold was applied to the time-series intensity data just above the background fluorescence levels at the initial time points. The time delay of the fluorescence response (*t*) was determined as the first time point when the intensity values of three neighboring time points were all greater than the threshold. The data (*t* and *d*) were then fitted to eq 3-4 using the in-built MATLAB algorithms *lsqcurvefit* and *fitnlm*, where *q*_*i*_ was set to 4, to determine the front advancement coefficient *D*_q_. The diffusion PDE was simulated as for the lawn assay simulations, with further details provided in the Supporting Information (Figure S6).

### Liquid Culture Fluorescence Assays

MK01 cells electroporated with the desired plasmids were grown shaking at 37 °C to the midexponential phase (OD_600_ 0.4–0.6). The cells were transferred to a 96-well plate, and the relevant inducer(s) dissolved in 2xYT medium were added. The total well volume was 150 μL. Fluorescence and absorbance at 600 nm were measured with the Tecan SPARK plate reader over 18 h. Settings for GFP: ex. 495/25 nm, em. 525/25 nm, gain 25. Settings for mCherry: ex. 625/25 nm, em. 625/25 nm, gain 50, Z-position 19200 μm. Shaking at 150 rpm, 2 mm.

The data were analyzed in MATLAB R2022a. The final fluorescence was subtracted from the background 2xYT-only readings. The data were divided by the absorbance wherever this is mentioned in the figures. The data was generally plotted as a function of the inducer concentration and fitted with Hill functions as shown (eq 2). The parameter α (background expression level) was calculated directly as the smallest fluorescence measurement. The *V*_m_, *K*_m_, and *n* parameters were then fitted using the MATLAB least-squares fitting function *lsqcurvefit*.

### Stripe Formation Assays

Single-well plates were set up with 12 mL of cell-agar mix. Cells containing the reporter plasmid with the dual-input promoter HC1–5 were grown to an OD600 of 0.6–0.8 and diluted 1:10 in warm molten agar (1.4% w/V) with antibiotics, ensuring that the agar temperature did not exceed 42 °C. The mixture was poured rapidly for the agar not to solidify. The final agar concentration was 1.3%. The agar was allowed to cool and solidify before the diffusers. A 2 μL droplet containing pC and OC6 at the desired concentrations (see Figures) was applied to the center of the plate and incubated at 37 °C overnight. For crescent experiments, two droplets were applied at the specified distances, whereas for concave diamonds, five droplets were applied. Plates were imaged after 20 h with the Zeiss Observer microscope, as described above (5× objective).

The model was simulated in two steps. First, the diffusion PDE was solved with the Crank-Nicolson solver in 2*D* space, as described for the lawn assay, over a grid of 43 × 43 mm with a 1 mm spacing between adjacent points. The initial and boundary conditions and other parameters were as used for the lawn assay simulations. Next, the spatial concentration profiles were fed through dual activation-repression Hill functions to convert them to fluorescence. The Hill functions were fitted to the HC1–5 promoter dose–response data, induced with pC and repressed with OC6.

## Supporting Information Available

Data processing, model fitting, dose–response assays, loss of cell lawn responsivity, cellular induction delay, effect of agar concentration on diffusion rate, data for all tested hybrid promoter variants, and all DNA and plasmid sequences (PDF)
