# Supplementary material for: Engineering Tunable, Low Latency Spatial Computation with Dual Input Quorum Sensing Promoters
Source: ACS Synth Biol. 2024 May 23;13(6):1750–61. doi: 10.1021/acssynbio.4c00068 (PMC11197083; doi:10.1021/acssynbio.4c00068)
Supplement: Supplementary file 1 — sb4c00068_si_001.pdf [file sb4c00068_si_001.pdf]

Supporting Information

Engineering tuneable, low latency spatial computation with dual input quorum sensing promoters

Jure Tica°[1], Haobin Chen°[1], Shulei Luo[1], Manman Chen[1], Mark Isalan[1,2]*

1 Department of Life Sciences, Imperial College London, London, SW7 2AZ, UK.
2 Imperial College Centre for Synthetic Biology, Imperial College London, London, SW7 2AZ, UK.

° Contributed equally
* Corresponding author, m.isalan@imperial.ac.uk

1 Supplementary 1: Lawn Assay 3
1.1 Diffusion model fitting and data processing 3
1.2 Dose-response assays 4
1.3 Loss of lawn responsivity 5
2 Supplementary 2: Distance Assay 6
2.1 Measurement of the cellular response delay t_{cell} 6
2.2 Advancement of the threshold concentration 7
2.3 Effect of agar concentration on diffusion rate 9
3 Supplementary 3: Hybrid Promoter Screening 10
3.1 Repressible promoter variants 10
3.2 Dual-input promoter variants 12
4 Supplementary 4: Ring Pattern Formation 14
5 Supplementary 5: DNA Sequences 17
5.1 Part sequences 17
5.2 Full plasmid sequences 22
5.2.1 pET-Px 22
5.2.2 p15A-xR 24
5.2.3 pCC1R 25
6 References 28

1 Supplementary 1: Lawn Assay

1.1 Diffusion model fitting and data processing

The raw microscopy images of **Figure 1B** were processed with a moving average filter of 120 x 120 pixels, to remove shading artefacts from tiling and to reduce the noise. The size of the images was 2780 x 4160 pixels, covering an area of 48 x 72 mm. **Figure S1** shows the raw data, together with the smoothened data.

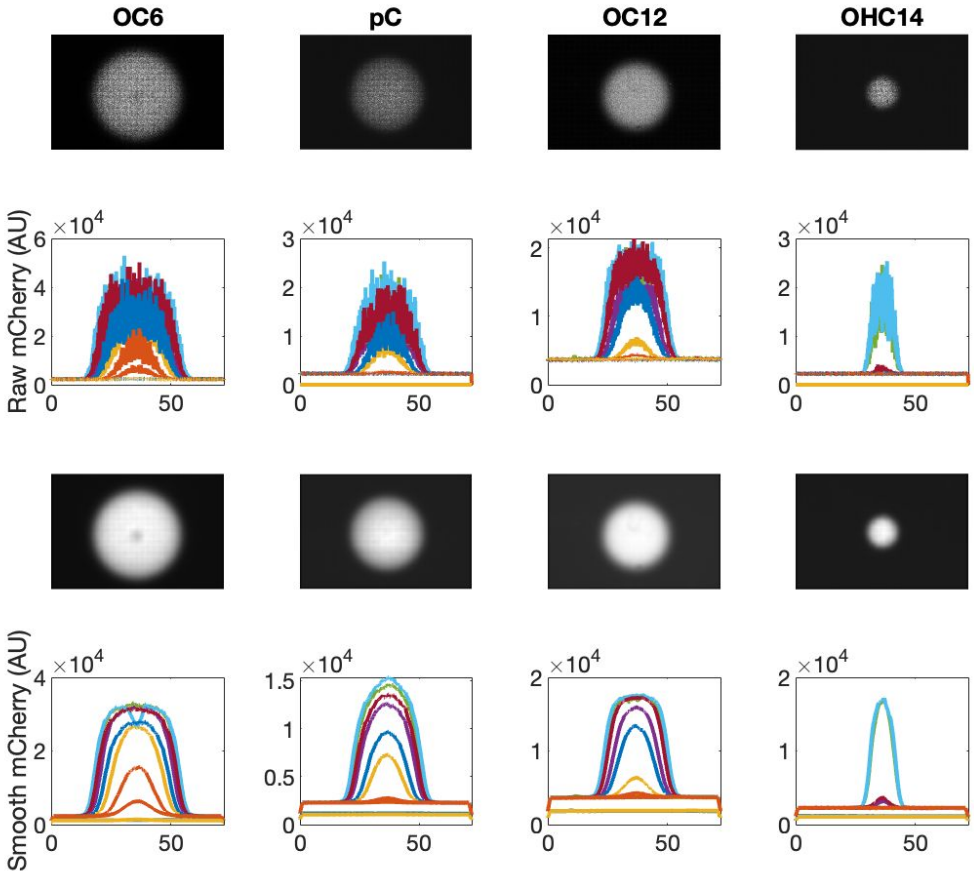

Figure S1: microscopy data smoothing with a moving average filter. A) Raw, unsmoothened data. B) Smoothened data. The display range for the images was adjusted to be between the minimum and maximum pixel intensity values for display purposes only.

1.2 Dose-response assays

Cells were suspended in 2xYT agar (1.4% w/V) and induced with spatially homogenous concentrations of diffusers. Cells harbouring the mCherry reporter constructs together with the Plux, Prpa, Plas and Pcin promoters were treated with OC6, pC, OC12 and OHC14, respectively. The fluorescence was measured with a microscope 16 hours after induction. The average image fluorescence was plotted against inducer concentration, for three biological replicates (**Figure S2**). This data was used to map inducer concentration to fluorescence in the diffusion models.

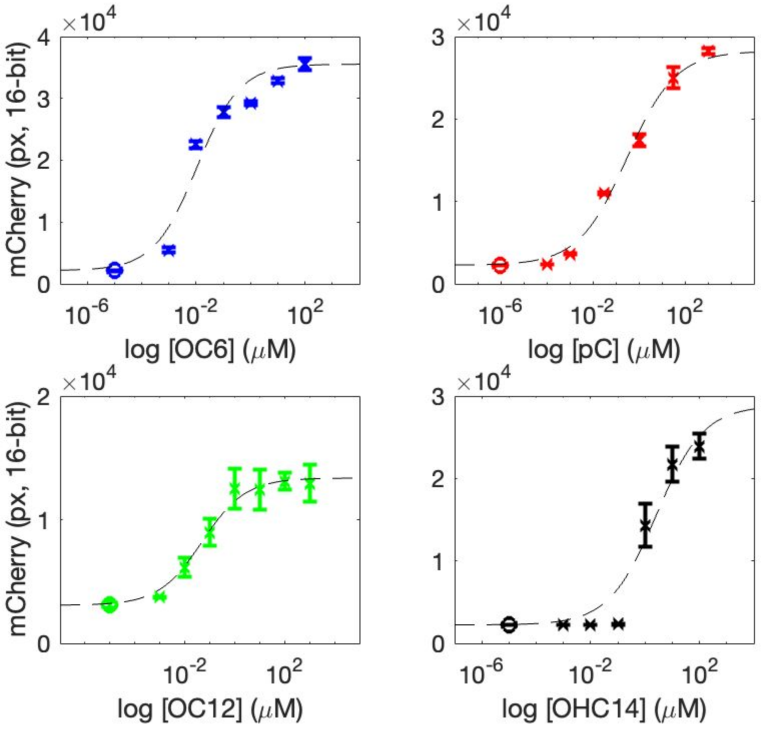

Figure S2: dose-response assays in a lawn of cells suspended in agar. Average fluorescence levels in the microscope images are plotted against inducer concentration. Activating Hill functions (Eq. 2) are fitted to the data, the parameters are listed in **Table S1**. Please note that the fluorescence levels for Plas/OC12 are not comparable with the other three datasets because a different exposure setting was used, see Methods.

Table S1: Hill parameters obtained by fitting to dose-response data.

Parameter	**OC6**	**pC**	**OC12**	**OHC14**
b	2.16e3	2.27e3	3.08e3	2.22e3
Vm	3.23e4	2.53e4	1.02e4	2.44e4
n	0.5	0.5	0.5	0.5
Km	9.1e-3	2.6e-1	4.9e-2	4.7e0

1.3 Loss of lawn responsivity

The reporter cells were plated as a lawn in an agar (1.4% w/V) suspension, incubated at 37 °C and induced with a delay between 0 and 10 hours. Fluorescence was measured at the 24 hour timepoint. The fluorescence response was allowed to develop fully for all the conditions. A gradual decline in the fluorescence response was observed with an increasing delay (**Figure S3**). The decline in fluorescence was fitted with linear functions (OC6 $R^2 = 0.68$, F-stat. vs. constant model $p < 0.1$; OHC14 $R^2 = 0.92$, F-stat. $p << 0.01$). This delay effect was more pronounced for the Pcin response to OHC14, where the system stopped responding after 11.5 hours. The Plux/OC6 system stopped responding after 19.2 hours. Overall, the results show that as they age, cells become less sensitive to the inducer. Similar findings are reported in other studies[1].

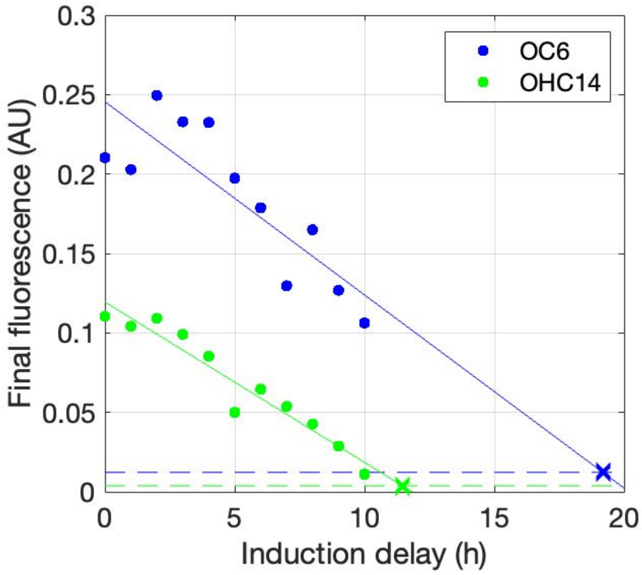

Figure S3: gradual decline of fluorescence response with a delay in induction. The response of the Plux/OC6 (blue) and Pcin/OHC14 (green) system declines with increasing delay between plating and induction. The filled dots are the average fluorescence intensities of microscope images collected at the 24 hour timepoint. The dashed lines are the background fluorescence intensities for the uninduced conditions at 24 hours. The crosses are the intersections between the fitted lines and the background intensity, marking the time when the cells stop responding (19.2 h for OC6, 11.5 h for OHC14).

2 Supplementary 2: Distance Assay

2.1 Measurement of the cellular response delay t_{cell}

The distance assay relies on measuring the delay between inducer droplet application and fluorescence expression. This delay includes the time needed for the diffuser to travel and reach the cells (diffusive delay, t_{diff}), and the time needed for the cell to respond once the diffusor has reached (cellular delay, t_{cell}). The relationship between distance and diffusive delay was used to calculate the diffusion rate of the molecules, whereas the cellular delay was treated as a constant as is inferred below.

The cellular delay was quantified by growing a small droplet of cells on top of agar containing a known concentration of spatially homogenous inducer. In this experiment t_{diff} is zero, allowing us to measure t_{cell} in isolation. This experiment was performed with the Prpa/pC system. It was assumed that similar laws also hold for the OC6/Plux, OC12/Plas and OHC14/Pcin systems.

Fluorescence measurements were taken every 5 minutes with a microscope, and average image fluorescence was plotted against time (**Figure S4A**). The final fluorescence was plotted against concentration and fitted with a Hill function (Fig SX). An arbitrary threshold was set to be just above the background fluorescence for each induction condition, and the time point at which the curves crossed this threshold was calculated and plotted against inducer concentration (**Figure S4B**). The data shows that t_{cell} does not change significantly across the effective diffusor concentration range (10^{-3} µM – 10^{3} µM). The fitted linear model is not significantly different from a constant model (F-stat. = 3.09; p = 0.177): t_{cell} can thus be treated as a constant.

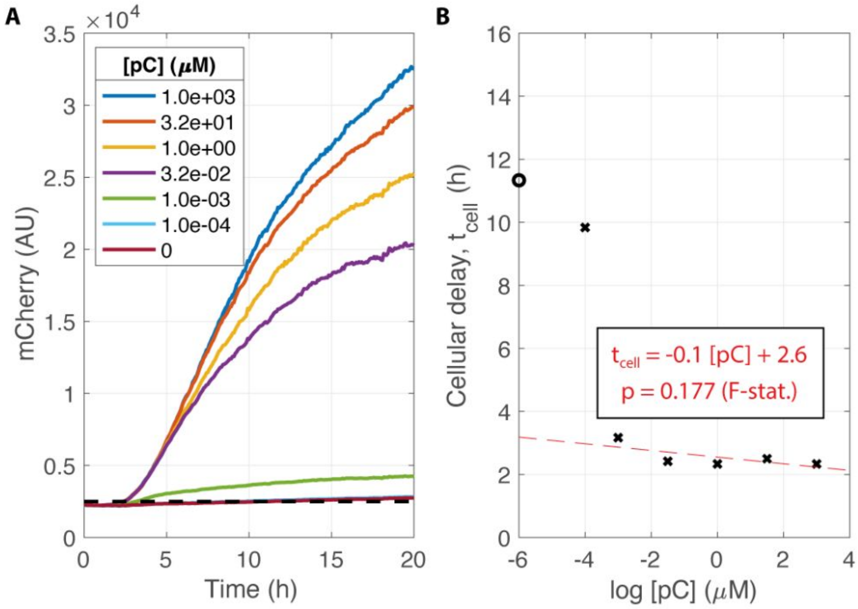

Figure S4: cellular response delay t_{cell}. A) mCherry fluorescence evolution in time for different concentrations of inducer pC. The time at which the curves cross the black dashed line is the cellular

delay (t_{cell}), plotted in B) as a function of pC concentration, and is a constant across the effective inducer concentration range.

2.2 Advancement of the threshold concentration

At the centre of the model fitting strategy for the distance assay are simulations of the diffusion PDE (Eq. 1). A typical solution of the diffusion equation is shown in **Figure S5A**. The coloured curves show the concentration of the diffuser in space for the successive timepoints of the simulation. A cross-section of a 2D simulation is plotted in one spatial dimension. The black horizontal line denotes the chosen threshold concentration c_t. The advancement of the location of the threshold concentration front is plotted in **Figure S5B**. The quadratic function of Eq. 4, describing the displacement of the diffusing molecule in time, fits the data well.

The advancement of the diffusing front is largely dependent on the diffusion rate D_X and on the ratio between the initial and threshold concentrations c_0/c_t. Scaling both c_0 and c_t by the same factor does not affect the shape of the quadratic relationship of **Figure S5B**. What matters is how the initial stimulus compares to the front concentration in relative terms.

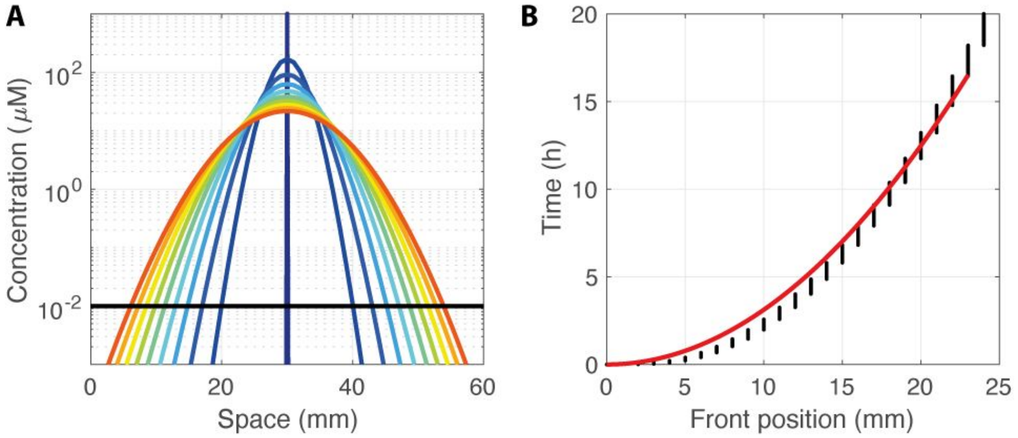

Figure S5: advancement of the threshold concentration in the diffusion PDE. A) A solution to the diffusion PDE from an initial condition c_0 of 10^3 µM with radius of 1 mm, D_X = 1 mm^2/h, over a period of 20 hours. The black horizontal line denotes the threshold concentration c_t of 10^{-2} µM. The points at the intersections between the coloured lines and the black line are the locations of the diffusing front at the c_t concentration at the respective timepoints. B) Plots the advancement of the c_t front in time. A quadratic relationship (Eq. 4) fits the data well (red curve).

The simulation of the diffusion PDE was repeated over a grid of D_X and c_0/c_t parameters. D_X was initialised in the interval [0.1, 2] mm^2/h, whereas c_0/c_t was initialised in [10^3, 10^9] µM. The simulations were performed over a 50 x 50 grid of logarithmically spaced points. The simulations were performed over a discretised grid of 60 x 60 points, where the size of the spatial domain was 60 mm x 60 mm. The initial condition c_0 was fixed to 10^3 µM, with a radius of 1 mm. The threshold concentration c_t was changed according to the value of the c_0/c_t parameter. The advancement of the c_t front is plotted in time in **Figure S6** for five values of D_X and c_0/c_t. The front advances more rapidly both when diffusion rate D_X is increased, and when the front concentration c_t is decreased. Hence, both the diffusion and kinetics influence the spatial response to the inducer, fluorescence in our case. Quadratic functions of Eq. 4 fit all the simulations well.

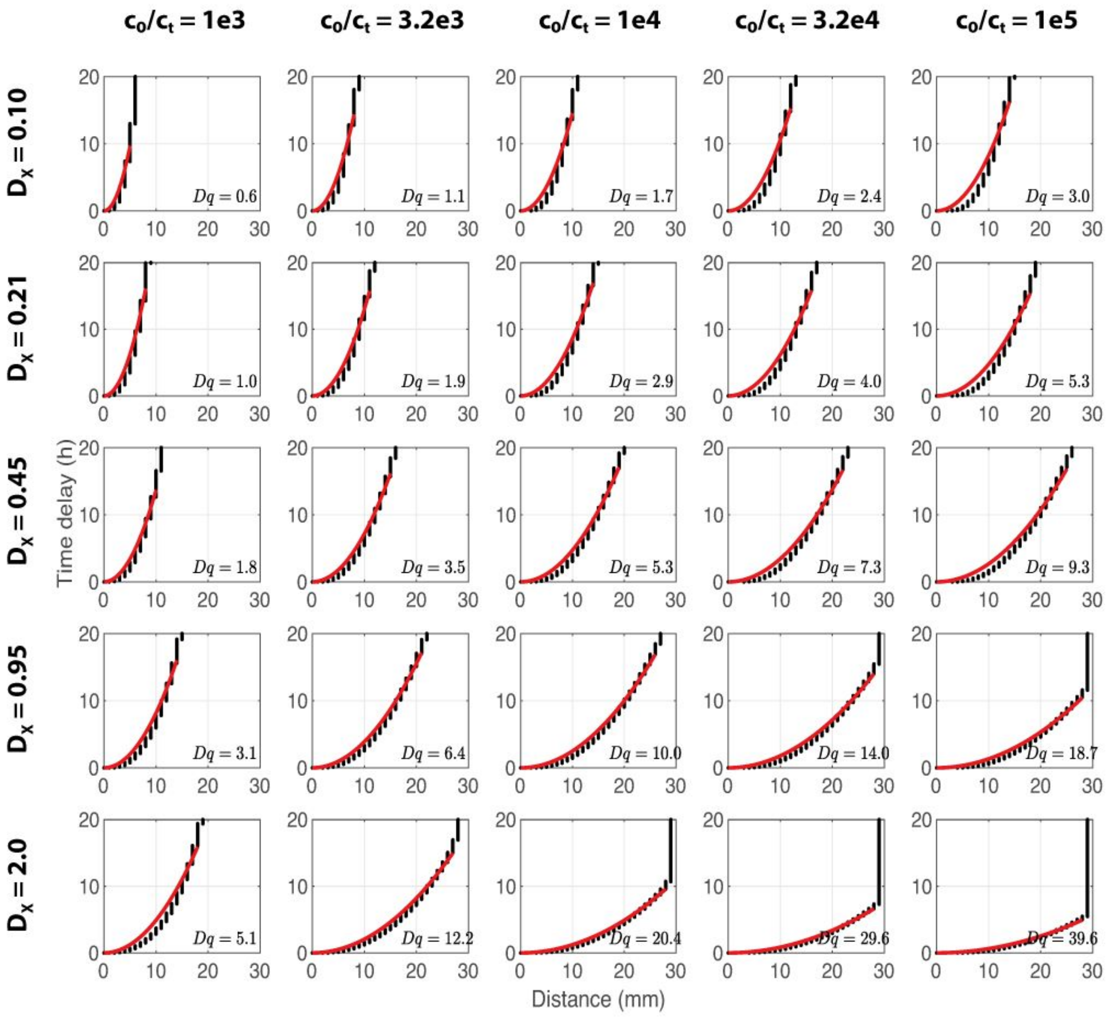

Figure S6: advancement of the threshold concentration front. The black points are derived from diffusion PDE simulations, by tracking the advancement of the c_t concentration front in time and space. Both diffusion rate D_X and threshold concentration c_t affect the dynamics of front advancement.

2.3 Effect of agar concentration on diffusion rate

Agar concentration is inversely proportional to diffusion rate. Diffusion of urea slows by 36% when agar concentration is changed from 0.8 to 5.15%[2]. Similarly, diffusion rate of glycerin drops by 33% when increasing agar concentration from 2 to 6%[2]. We tested diffusion of OC6 in 0.4%, 1.4% and 2.4% agar using the distance-based assay (**Figure S7**). The diffusion of OC6 decreased by 40% when increasing agar concentration from 0.4% to 2.4%.

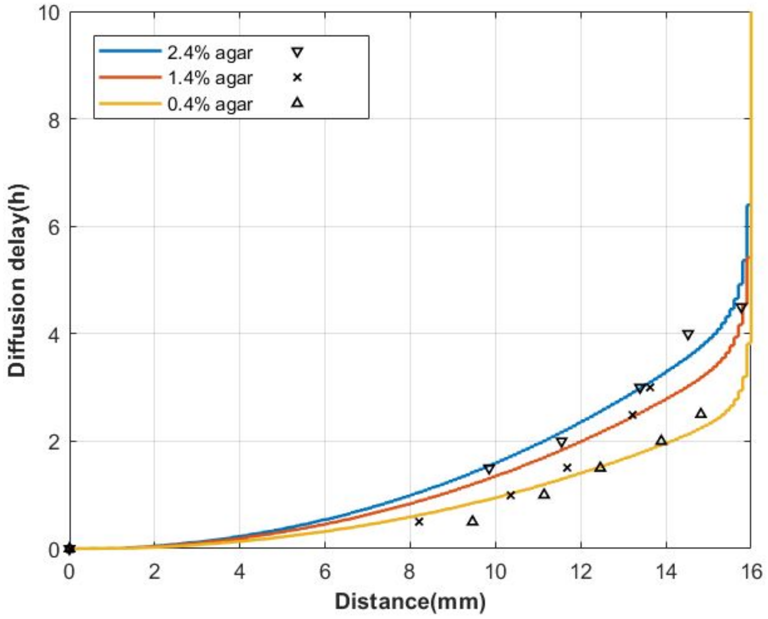

Figure S7: effect of agar concentration on diffusion rate. Experimental results are shown with dots. The best fit models are shown with lines: these were obtained by simulating the diffusion PDE (Eq. 1) and plotting the advancement of the threshold concentration that leads to the minimal sensing event in space and time. The initial concentration of OC6 was 10^4 µM. The diffusion rates of OC6 in 2.4%, 1.4%, and 0.4% agar are 2.0, 2.4, and 3.4 mm^2/h, respectively. While these values are largely consistent with those reported in the main paper, the small differences are likely due to small changes in the experimental protocols. This data had the cellular delay t_{cell} subtracted for each agar concentration individually, the plot only shows diffusive delay t_{diff}.

3 Supplementary 3: Hybrid Promoter Screening

3.1 Repressible promoter variants

Promoters that are repressed by a single quorum sensing signal were designed first. The operators (**Error! Reference source not found.**) were placed immediately downstream a constitutive J23106 promoter. Some designs immediately showed strong repression (e.g. J23106-luxO), others showed intermediate repression (e.g. J23106-rpaO), and one showed no repression (e.g. J23106-cinO), see Fig. 3B.

To increase the repression levels multiple copies of the operators were placed in tandem downstream J23106. One, two and three copies were used for lasO. One, four and seven copies were tested for rhlO. The results were not as expected, where two operator copies showed a weaker repression compared to a single copy (**Figure S8**). Surprisingly, three or more copies reversed the effect and showed an induction rather than repression. This strategy of increasing repression strength was therefore abandoned.

Repression for single and double lasO variants was observed for both OC12 and OC16, consistent with observations that Prhl can sense multiple long chain AHLs[3].

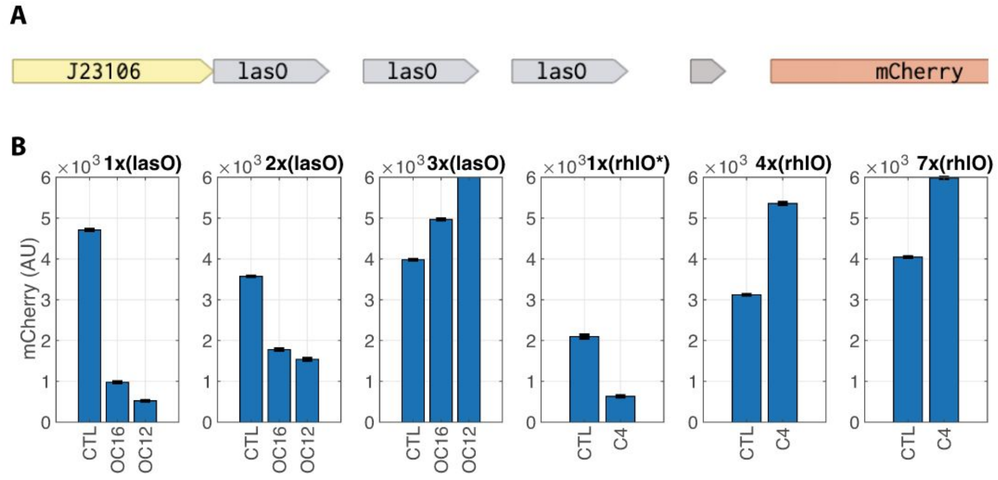

Figure S8: constitutive promoters J23106 combined with one or more quorums sensing operators. A) One or more operators lasO and rhlO were placed downstream J23106 with short spacer sequences in between, followed by an RBS (darker grey) and the mCherry reporter gene. B) mCherry fluorescence after addition of OC16, OC12 for lasO variants, and C4 for rhlO variants at 100 µM.

To debug the non-functional J23106-cinO variant, we placed short segments of the Pcin promoter downstream J23106 to test their ability to repress. Using this approach, we obtained two functional variants, v1 and v2 (**Figure S9**). The v1 variant is weaker, but exhibits slightly stronger repression levels. On the other hand, the v2 variant preserves the constitutive activity of the J23106 promoter better, but displays slightly lower, even though comparable, fold-repression.

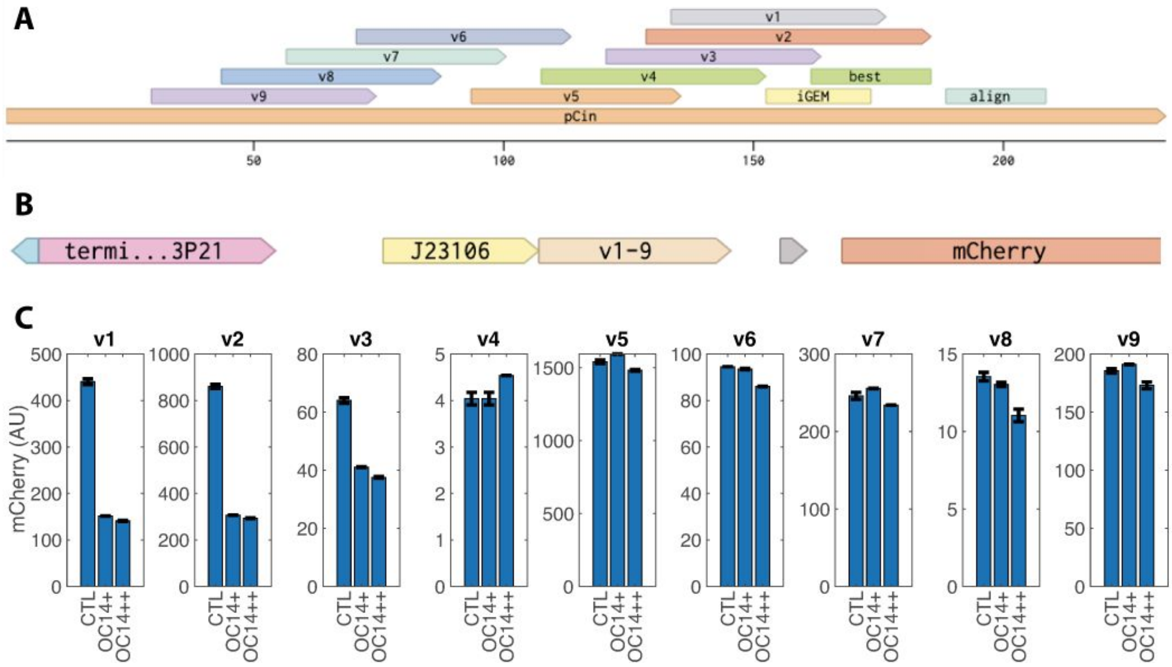

Figure S9: placing short segments of the Pcin promoter downstream J23106. A) The tested Pcin fragments (v1 – v9) are aligned to the Pcin promoter. The operator site identified by iGEM teams and Meyer et al. (2019) is labelled with 'iGEM'. The best site as determined by testing v1 – v9 is labelled with 'best'. The luxO was aligned with the Pcin sequence in the site labelled with 'align'. B) The v1 – v9 fragments were cloned downstream of J23106, the short RBS is shown in dark grey followed by the mCherry reporter gene. C) mCherry fluorescence levels. The v1, v2 and v3 promoters were successfully repressed by adding OHC14 at 10 µM (+) and 100 µM (++).

Table S2: operator DNA sequences.

Operator	Sequence
rhlO	TCCTGTGAAATCTGGCAGTT
luxO	ACCTGTAGGATCGTACAGGT
rpaO	ACCTGTCCGATCGGACAGTA
lasO	AACTAGCAAATGAGATAGAT
cinO (v1)	GCTCTGATCCCCCTCATCTGGGGGGGCCTATCTGAGGGAATTT
cinO (v2)	GACATGCTCTGATCCCCCTCATCTGGGGGGGCCTATCTGAGGGAATTTCCGATCCGG

3.2 Dual-input promoter variants

The first series of dual-input promoter variants (HC1 series) was based on the designs put forward by Zucca et al. (2015) and Du et al. (2020), where the activating operator site is placed immediately upstream the promoter, whereas the repressing operator is placed immediately downstream or in the core promoter region (**Figure S10**)[4,5]. The HC1-5 promoter is the best in the series and shows strong activation by pC and tight repression by OC6; no activation is seen in the presence of OC6 alone, indicating no cross-talk between the operators. The HC1-6 promoter lost all activity probably owing to the strong luxO site being placed in the core promoter region. HC1-7 is another promising design, activated by OC6 and repressed by pC; the repression could be further optimised by tuning the rpaO site. HC1-8 shows very low activity when activated by OC6, and cannot be repressed by pC. HC1-9 and HC1-10 show good activation by OC6, but cannot be repressed by OHC14, probably owing to a dysfunctional cinO (**Figure S9**, iGEM site[6], no activity seen in any of the designs), and could be optimised by using the cinO-v1 site. Overall, this shows that operators perform better in the distal, downstream site (rather than in the core region). Furthermore, the data shows that this architecture yields functional dual-input promoters; these could be further optimised by tuning the operator sites to increase their strength.

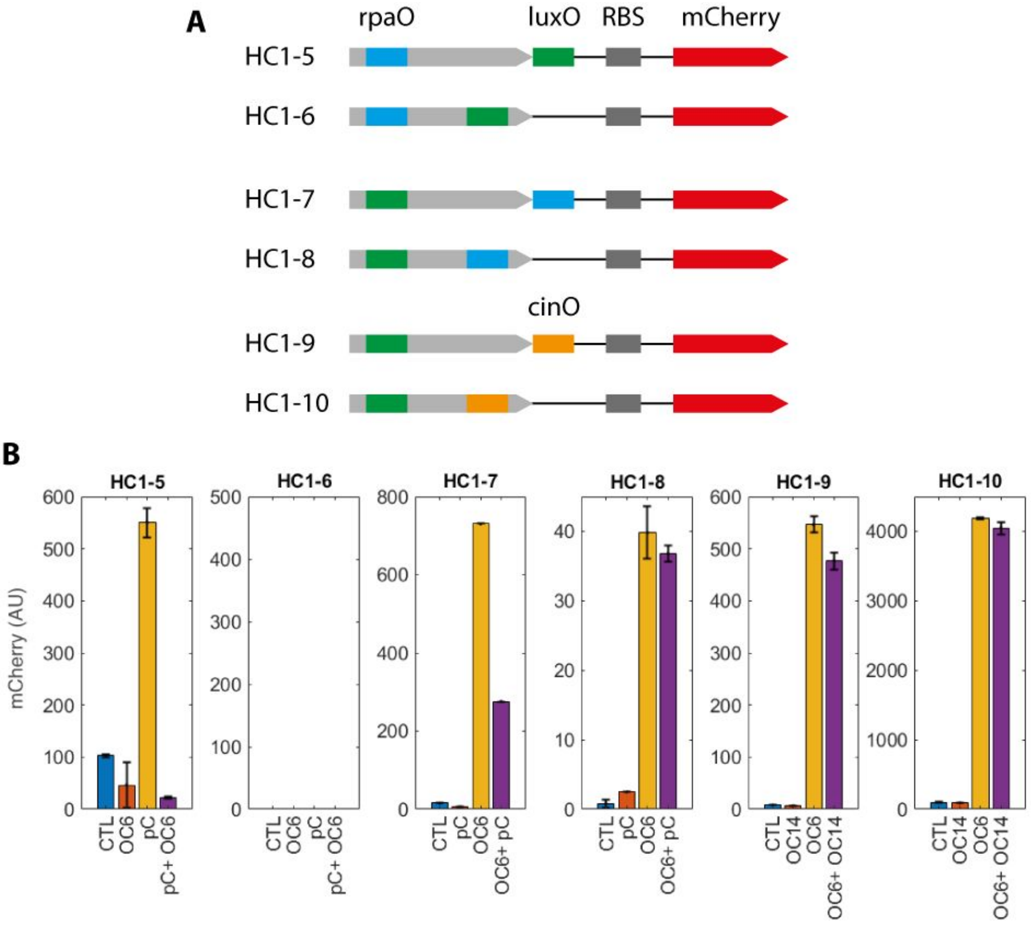

Figure S10: HC1 series of dual-input promoters. A) DNA architecture of the tested designs. B) mCherry fluorescence levels. The HC1-5 and HC1-7 promoters show good performance with induction and repression by their respective regulators. Concentration of inducers: 10 µM.

The second series of dual-input promoters (HC2 series) involved placing operators into the Pcin promoter (iGEM R0078). Operators rpaO and luxO were placed in the core region and immediately downstream (**Figure S11**). The HC2-1 and HC2-2 promoters can be activated by OHC14 but show weak repression by rpaO; this is consistent with the weak repression levels that rpaO achieves when placed downstream J23106 (Fig. 3B). The HC2-3 promoter is the best in this series and shows good induction by OHC14 and repression by OC6. The HC2-4 promoter lost all activity, owing to the strong luxO site being placed inside the promoter. These promoters show promising results, but the strength of repression can be further optimised by tuning the operator sequences.

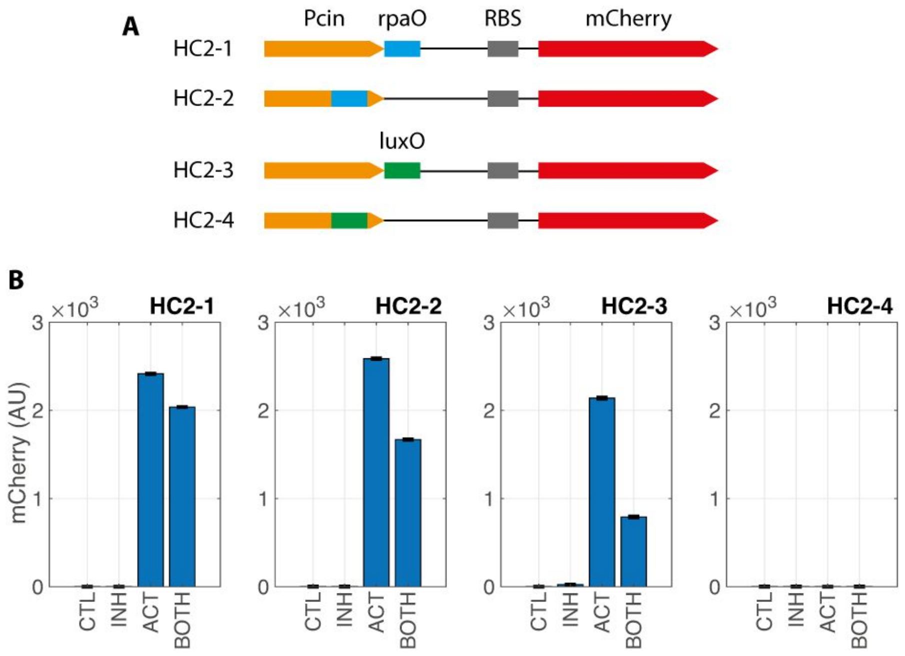

Figure S11: HC2 series of dual-input promoters. A) DNA architecture of the tested designs. B) mCherry fluorescence levels. The HC2-2 and HC2-3 promoters show good performance with induction and repression by their respective regulators, but can be improved by strengthening the repression arm. Concentration of inducers: 10 µM.

4 Supplementary 4: Ring Pattern Formation

The model for ring formation consisted of two steps. The first step involved solving the diffusion PDE for both the activator and inhibitor species and their diffusion rates D_X. The result of one of these simulations is shown in **Figure S12**, where the inducers are spotted in the centre of the spatial domain and diffuse towards the periphery. The parameters in this example are D_{act} = 0.6 and D_{inh} = 0.9, consistent with the lawn diffusion rates of pC and OC6, respectively (Table 1).

The simulation results show that the ring forms and expands over time, due to the gradual diffusion of the molecules outwards (**Figure S12E**). This is however not observed in the experiments, where a ring of fixed diameter forms on the lawn of cells. This behaviour is analogous to the temporal evolution of mCherry fluorescence of Fig. 1C, where the bell-shaped fluorescence distribution does not broaden with time, even though the molecules continue to diffuse outwards. The cellular lawn gives a snapshot of diffuser concentrations at a particular timepoint, which occurs sometime between t_0 and the time when the response reaches full induction, at about 10 hours (**Figure S13B**). The 10 hour timepoint was chosen when modelling the ring systems (Fig. 4C). This is also likely related to the dynamics of the loss of lawn responsivity, which also occurs at the 10 hour timepoint for the OHC14 system (**Figure S3**).

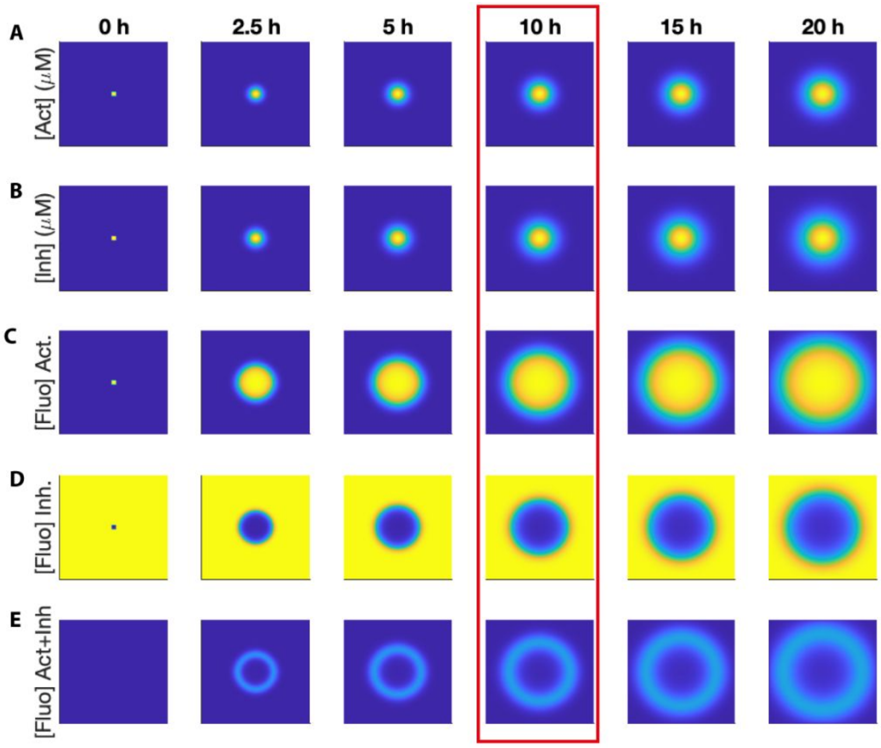

Figure S12: simulation of ring formation in time. A) Activator concentration in space, as obtained from the diffusion PDE simulation. B) Inhibitor concentration in space. C) Cellular response to the activator, obtained after feeding the activator concentrations through a Hill function fitted to the dose-response data of the HC1-5 promoter. D) Cellular response to the inhibitor over a fully activated lawn of cells, obtained after feeding the inhibitor concentrations through a Hill function of promoter HC1-5. The activator concentration was set to a value that produces full activation in the absence of

the inhibitor. E) Cellular response to both the activator and inhibitor over a lawn of cells with the HC1-5 promoter. The timepoint of 10 h is highlighted in red because it was selected for the model simulations in Fig. 4. The size of the images is 43 mm x 43 mm, same as for Fig. 4C.

The second step of the modelling process for the ring patterns involved fitting a dual-input Hill function (Eq. S1) to the dose-response data of the underlying promoter, HC1-5 in our case. This model captures the data well (**Figure S13**). The best fit parameters are listed in **Table S3**.

$$F_{dual}([pC],[OC_6]) = \left(\alpha_a + \frac{V_a}{1+\left(\frac{K_a}{[pC]}\right)^{n_a}}\right)\left(\alpha_i + \frac{V_i}{1+\left(\frac{[OC_6]}{K_i}\right)^{n_i}}\right) \quad \text{S1}$$

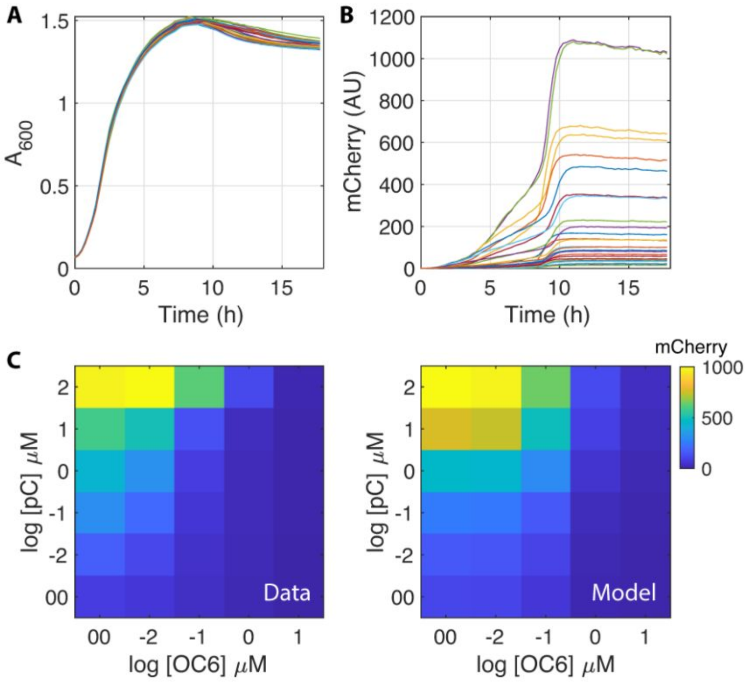

Figure S13: HC1-5 dose-response data and dual-input Hill function model fit. A) Timeseries absorbance measurements at 600 nm. Normal cell culture growth is detected for all inducer combinations. The system shows no toxicity or burden at any of the mCherry expression levels. B) Timeseries mCherry measurements, where a range of induction levels is observed for the different inducer combinations. The fluorescence response is fully developed approximately 11 hours after induction. The shape of the curves and the triphasic growth of fluorescence is likely due to the slow and multi-step maturation of the fluorophore[7]. C) Final mCherry levels plotted against pC and OC6 concentration, where 00 are the no-inducer controls. D) Dual-input Hill model reproduces the experimental data in C).

Table S3: fitted dual-input Hill function parameters.

Par (activation)	Value
α_a	30
V_a	980
K_a	0.15
n_a	1.2

Par (inhibition)	Value
α_i	100
V_i	1000
K_i	4.2
n_i	0.5

5 Supplementary 5: DNA Sequences

In a nutshell, all the experiments were conducted by transforming cells with a pET reporter plasmid containing an mCherry reporter gene expressed under a promoter of interest, together with the pCC1R plasmid containing constitutively expressed receptor genes rpaR and cinR, which bind to the inducers and regulate the promoter activity. An additional p15A plasmid harbouring the luxR, lasR and rhlR genes was used when needed.

The pET and p15A are low-to-medium copy plasmids, whereas pCC1 (CopyControl plasmid) is close to single copy.

5.1 Part sequences

Table S4: quorum sensing inducers used in this study, dissolved in DMSO at 10 to 50 mM.

Abbreviation	Full name	Receptor gene	Catalogue number
OHC14	N-(3-Hydroxytetradecanoyl)-DL-homoserine lactone	CinR	Sigma-Aldrich 51481
pC	N-(p-Coumaroyl)-L-homoserine lactone	RpaR	Sigma-Aldrich 07077
OC6	N-(β-ketocaproyl)-L-Homoserine lactone	LuxR	Cayman Chemical 10011207
OC12	N-(3-Oxododecanoyl)-L-homoserine lactone	LasR	Cayman Chemical 10007895
C4	N-butyryl-L-Homoserine lactone	RhlR	Sigma-Aldrich SML3427

Table S5: genetic part sequences derived from other studies.

Name	Sequence	Regulation	Source
Plux	ACCTGTAGGATCGTACAGGTTTACGCAAGAAAATG GTTTGTTACTTTCGAATAAA	OC6 +	[4]
Prpa	ACCTGTCCGATCGGACAGTTTTACGCAAGAAAATG GTTTGTTACTTTCGAATAAA	pC +	[4]
Pcin	CCCTTTGTGCGTCCAAACGGACGCACGGCGCTCTAA AGCGGGTCGCGATCTTTCAGATTCGCTCCTCGCGCT TTCAGTCTTTGTTTTGGCGCATGTCGTTATCGCAAAA CCGCTGCACACTTTTGCGCGACATGCTCTGATCCCC CTCATCTGGGGGGGCCTATCTGAGGGAATTTCCGA	OHC14 +	[6]

	TCCGGCTCGCCTGAACCATTCTGCTTTCCACGAACTTGAAAACGCT		
Prhl	TCCTGTGAAATCTGGCAGTTTTACGCAAGAAAATGGTTTGTTACTTTCGAATAAA (not used here)	C4 +	[4]
Plas	AACTAGCAAATGAGATAGATTTACGCAAGAAAATGGTTTGTTACTTTCGAATAAA	OC12 +	[4]
LuxR	ATGAAGAACATCAATGCGGACGACACCTACCGTATCATCAATAAGATCAAAGCGTGCCGCAGCAATAATGACATCAACCAGTGCCTGAGCGACATGACCAAGATGGTGCACTGCGAGTACTATCTGCTGGCGATCATTTACCCGCACAGCATGGTTAAAAGCGACATCAGCATTCTGGATAACTATCCGAAGAAATGGCGTCAGTACTATGACGATGCGAACCTGATCAAGTACGACCCGATTGTGGATTATAGCAACAGCAACCACAGCCCGATCAACTGGAACATTTTCGAGAACAACGCGGTGAACAAGAAAAGCCCGAACGTTATCAAGGAAGCGAAAACCAGCGGTCTGATCACCGGCTTCAGCTTTCCGATTCACACCGCGAACAACGGTTTCGGCATGCTGAGCTTTGCGCACAGCGAAAAAGACAACTACATCGATAGCCTGTTTCTGCACGCGTGCATGAACATCCCGCTGATTGTGCCGAGCCTGGTTGACAACTATCGTAAGATCAACATTGCGAACAACAAAAGCAACAACGATCTGACCAAGCGTGAGAAAGAATGCCTGGCGTGGGCGTGCGAGGGCAAGAGCAGCTGGGATATCAGCAAAATTCTGGGCTGCAGCGAACGTACCGTTACCTTCCACCTGACCAACGCGCAGATGAAGCTGAACACCACCAACCGTTGCCAAAGCATCAGCAAAGCGATCCTGACCGGTGCGATTGACTGCCCGTACTTCAAGAACTAATAA	OC6 receptor	*V. fischeri*
RhlR	ATGCGCAACGATGGCGGCTTTCTGCTGTGGTGGGATGGCCTGCGCAGCGAAATGCAGCCGATTCATGATAGCCAGGGCGTGTTTGCGGTGCTGGAAAAAGAAGTGCGCCGCCTGGGCTTTGATTATTATGCGTATGGCGTGCGCCATACCATTCCGTTTACCCGCCCGAAAACCGAAGTGCATGGCACCTATCCGAAAGCGTGGCTGGAACGCTATCAGATGCAGAACTATGGCGCGGTGGATCCGGCGATTCTGAACGGCCTGCGCAGCAGCGAAATGGTGGTGTGGAGCGATAGCCTGTTTGATCAGAGCCGCATGCTGTGGAACGAAGCGCGCGATTGGGGCCTGTGCGTGGGCGCGACCCTGCCGATTCGCGCGCCGAACAACCTGCTGAGCGTGCTGAGCGTGGCGCGCGATCAGCAGAACATTAGCAGCTTTGAACGCGAAGAAATTCGCCTGCGCCTGCGCTGCATGATTGAACTGCTGACCCAGAAACTGACCGATCTGGAACATCCGATGCTGATGAGCAACCCGGTGTGCCTGAGCCATCGCGAACGCGAAATTCTGCAGTGGACCGCGGATGGCAAAAGCAGCGGCGAAATTGCGATTATTCTGAGCATTAGCGAAAGCACCGTGAACTTTCATCATAAAAACATTCAGAAAAAATTT	C4 receptor	*P. aeruginosa*

	GATGCGCCGAACAAAACCCTGGCGGCGGCGTATGC GGCGGCGCTGGGCCTGATTTAATAA		
LasR	ATGGCGCTGGTGGATGGCTTTCTGGAACTGGAACG CAGCAGCGGCAAACTGGAATGGAGCGCGATTCTGC AGAAAATGGCGAGCGATCTGGGCTTTAGCAAAATT CTGTTTGGCCTGCTGCCGAAAGATAGCCAGGATTAT GAAAACGCGTTTATTGTGGGCAACTATCCGGCGGC GTGGCGCGAACATTATGATCGCGCGGGCTATGCGC GCGTGGATCCGACCGTGAGCCATTGCACCCAGAGC GTGCTGCCGATTTTTTGGGAACCGAGCATTTATCAG ACCCGCAAACAGCATGAATTTTTTGAAGAAGCGAG CGCGGCGGGCCTGGTGTATGGCCTGACCATGCCGC TGCATGGCGCGCGCGGCGAACTGGGCGCGCTGAG CCTGAGCGTGGAAGCGGAAAACCGCGCGGAAGCG AACCGCTTTATGGAAAGCGTGCTGCCGACCCTGTG GATGCTGAAAGATTATGCGCTGCAGAGCGGCGCGG GCCTGGCGTTTGAACATCCGGTGAGCAAACCGGTG GTGCTGACCAGCCGCGAAAAAGAAGTGCTGCAGTG GTGCGCGATTGGCAAAACCAGCTGGGAAATTAGCG TGATTTGCAACTGCAGCGAAGCGAACGTGAACTTTC ATATGGGCAACATTCGCCGCAAATTTGGCGTGACCA GCCGCCGCGTGGCGGCGATTATGGCGGTGAACCTG GGCCTGATTACCCTGTAATAA	OC12 receptor	*P. aeruginosa*

Table S6: genetic sequences of the repressible promoters assembled and tested here.

Name	Sequence	Regulation
J23106-luxO	TTTACGGCTAGCTCAGTCCTAGGTATAGTGCTAGC**ACC TGTAGGATCGTACAGGT**	OC6 –
J23106-rpaO	TTTACGGCTAGCTCAGTCCTAGGTATAGTGCTAGC**ACC TGTCCGATCGGACAGTT**	pC –
J23106-cinO (J250_fw4)	TTTACGGCTAGCTCAGTCCTAGGTATAGTGCTAGC**GCT CTGATCCCCCTCATCTGGGGGGGCCTATCTGAGGGAA**	OHC14 –
J23106-lasO	TTTACGGCTAGCTCAGTCCTAGGTATAGTGCTAGC**AAC TAGCAAATGAGATAGAT**	OC12 –
J23106-rhlO	TTTACGGCTAGCTCAGTCCTAGGTATAGTGCTAGC**TCC TGTGAAATCTGGCAGTT**	C4 –

Table S7: genetic sequences of the hybrid promoters assembled and tested here.

Name	Sequence	Regulation

P_HC1-5	**ACCTGTCCGATCGGACAGTT**TTACGCAAGA<br>AAATGGTTTGTTACTTTCGAATAA**ACCTGTA**<br>**GGATCGTACAGGT**	pC +; OC6 –
P_HC1-6	**ACCTGTCCGATCGGACAGTT**TTACG**ACCTG**<br>**TAGGATCGTACAGGT**ACTTTCGAATAAA	pC +; OC6 –
P_HC1-7	**ACCTGTAGGATCGTACAGGT**TTACGCAAGA<br>AAATGGTTTGTTACTTTCGAATAA**ACCTGTC**<br>**CGATCGGACAGTA**	OC6 +; pC –
P_HC1-8	**ACCTGTAGGATCGTACAGGT**TTACG**ACCTG**<br>**TCCGATCGGACAGTT**ACTTTCGAATAAA	OC6 +; pC –
P_HC1-9	**ACCTGTAGGATCGTACAGGT**TTACGCAAGA<br>AAATGGTTTGTTACTTTCGAATAAAGGGGG<br>GGCCTATCTGAGGGAA	OC6 +; OHC14<br>–
P_HC1-10	**ACCTGTAGGATCGTACAGGT**TTACGGGGG<br>GGCCTATCTGAGGGAATACTTTCGAATAAA	OC6 +; OHC14<br>–
P_HC2-1	CCCTTTGTGCGTCCAAACGGACGCACGGCGCTCTAAA<br>GCGGGTCGCGATCTTTCAGATTCGCTCCTCGCGCTTTC<br>AGTCTTTGTTTTGGCGCATGTCGTTATCGCAAAACCGC<br>TGCACACTTTTGCGCGACATGCTCTGATCCCCCTCATC<br>TGGGGGGGCCTATCTGAGGGAATTTCCGATCCGGCTC<br>GCCTGAACCATTCTGCTTTCCACGAACTTGAAAACGCT<br>**ACCTGTCCGATCGGACAGTA**	OHC14 +; pC –
P_HC2-2	CCCTTTGTGCGTCCAAACGGACGCACGGCGCTCTAAA<br>GCGGGTCGCGATCTTTCAGATTCGCTCCTCGCGCTTTC<br>AGTCTTTGTTTTGGCGCATGTCGTTATCGCAAAACCGC<br>TGCACACTTTTGCGCGACATGCTCTGATCCCCCTCATC<br>TGGGGGGGCCTATCTGAGGGAATTTCCG**ACCTGTC**<br>**CGATCGGACAGTA**CATTCTGCTTTCCACGAACTTG<br>AAAACGCT	OHC14 +; pC –
P_HC2-3	CCCTTTGTGCGTCCAAACGGACGCACGGCGCTCTAAA<br>GCGGGTCGCGATCTTTCAGATTCGCTCCTCGCGCTTTC<br>AGTCTTTGTTTTGGCGCATGTCGTTATCGCAAAACCGC<br>TGCACACTTTTGCGCGACATGCTCTGATCCCCCTCATC<br>TGGGGGGGCCTATCTGAGGGAATTTCCGATCCGGCTC<br>GCCTGAACCATTCTGCTTTCCACGAACTTGAAAACGCT<br>**ACCTGTAGGATCGTACAGGT**	OHC14 +; OC6<br>–

P_HC2-4	CCCTTTGTGCGTCCAAACGGACGCACGGCGCTCTAAAGCGGGTCGCGATCTTTCAGATTCGCTCCTCGCGCTTTCAGTCTTTGTTTTGGCGCATGTCGTTATCGCAAAACCGCTGCACACTTTTGCGCGACATGCTCTGATCCCCCTCATCTGGGGGGGCCTATCTGAGGGAATTTCCG**ACCTGTAGGATCGTACAGGT**CATTCTGCTTTCCACGAACTTGAAAACGCT	OHC14 +; OC6 –

5.2 Full plasmid sequences

Legend: Promoter Operator RBS Terminator Gene

5.2.1 pET-Px

Universal backbone for inducer sensing (Section 1 and 2) and promoter testing (Section 3) constructs, mCherry is used as reporter. The Px sequences are listed in Tables S3-S5.

TT L3S3P21 :: Px :: RBS BBa_B0034 :: mCherry_ASV :: TT rrnB-T1 :: AmpR :: pET ori

Grey highlighted sequence is removed in HC1-5 and HC1-7 constructs, consisting of 2 restriction sites.

CCAATTATTGAAGGCCTCCCTAACGGGGGGCCTTTTTTTGTTTCTGGTCTCCCGCTTAACGATCGTTGGCTGGGT
ACCXXXXXXXXXXXXXXXXXXXXXXXXXXXXXXXXXACGCGTTCTAGAGAAAGAGGAGAA
ATACTAGATGGTGAGCAAGGGCGAGGAGGATAACATGGCTATCATCAAGGAGTTCATGCGCTTCAAGGTGCACAT
GGAGGGCTCCGTGAACGGCCACGAGTTCGAGATCGAGGGCGAGGGCGAGGGCCGCCCCTACGAGGGCACCCAGAC
CGCCAAGCTGAAGGTGACCAAGGGTGGCCCCCTGCCCTTCGCCTGGGACATCCTGTCCCCTCAGTTCATGTACGG
CTCCAAGGCCTACGTGAAGCACCCCGCCGACATCCCCGACTACTTGAAGCTGTCCTTCCCCGAGGGCTTCAAGTG
GGAGCGCGTGATGAACTTCGAGGACGGCGGCGTGGTGACCGTGACCCAGGACTCCTCCCTGCAGGACGGCGAGTT
CATCTACAAGGTGAAGCTGCGCGGCACCAACTTCCCCTCCGACGGCCCCGTAATGCAGAAGAAGACTATGGGCTG
GGAGGCCTCCTCCGAGCGGATGTACCCCGAGGACGGCGCCCTGAAGGGCGAGATCAAGCAGAGGCTGAAGCTGAA
GGACGGCGGCCACTACGACGCTGAGGTCAAGACCACCTACAAGGCCAAGAAGCCCGTGCAGCTGCCCGGCGCCTA
CAACGTCAACATCAAGTTGGACATCACCTCCCACAACGAGGACTACACCATCGTGGAACAGTACGAACGCGCCGA
GGGCCGCCACTCCACCGGCGGCATGGACGAGCTGTACAAGAGGCCTGCTGCTAACGATGAAAACTACGCAgctAG
CgttTGATAATAGAGGCATCAAATAAAACGAAAGGCTCAGTCGAAAGACTGGGCCTTTCGTTTTATCTGTTGTTT
GTCGGTGAACGCTCTCCTGAGTAGGACAAATCCCTCGAGgacgtcaggtggcacttttcggggaaatgtgcgcgg
aacccctatttgtttatttttctaaatacattcaaatatgtatccgctcatgagacaataaccctgataaatgct
tcaataatattgaaaaaggaagagtatgagtattcaacatttccgtgtcgcccttattcccttttttgcggcatt
ttgccttcctgtttttgctcacccagaaacgctggtgaaagtaaaagatgctgaagatcagttgggtgcacgagt
gggttacatcgaactggatctcaacagcggtaagatccttgagagttttcgccccgaagaacgttttccaatgat
gagcacttttaaagttctgctatgtggcgcggtattatcccgtgttgacgccgggcaagagcaactcggtcgccg
catacactattctcagaatgacttggttgagtactcaccagtcacagaaaagcatcttacggatggcatgacagt
aagagaattatgcagtgctgccataaccatgagtgataacactgcggccaacttacttctgacaacgatcggagg
accgaaggagctaaccgcttttttgcacaacatgggggatcatgtaactcgccttgatcgttgggaaccggagct
gaatgaagccataccaaacgacgagcgtgacaccacgatgcctgcagcaatggcaacaacgttgcgcaaactatt
aactggcgaactacttactctagcttcccggcaacaattaatagactggatggaggcggataaagttgcaggacc
acttctgcgctcggcccttccggctggctggtttattgctgataaatctggagccggtgagcgtgggtctcgcgg
tatcattgcagcactggggccagatggtaagccctcccgtatcgtagttatctacacgacggggagtcaggcaac
tatggatgaacgaaatagacagatcgctgagataggtgcctcactgattaagcattggtaactgtcagaccaagt
ttactcatatatactttagattgatttaaaacttcatttttaatttaaaaggatctaggtgaagatcctttttga
taatctcatgaccaaaatcccttaacgtgagttttcgttccactgagcgtcagaccccgtagaaaagatcaaagg
atcttcttgagatcctttttttctgcgcgtaatctgctgcttgcaaacaaaaaaaccaccgctaccagcggtggt
ttgtttgccggatcaagagctaccaactctttttccgaaggtaactggcttcagcagagcgcagataccaaatac
tgtccttctagtgtagccgtagttaggccaccacttcaagaactctgtagcaccgcctacatacctcgctctgct

aatcctgttaccagtggctgctgccagtggcgataagtcgtgtcttaccgggttggactcaagacgatagttacc
ggataaggcgcagcggtcgggctgaacggggggttcgtgcacacagcccagcttggagcgaacgacctacaccga
actgagatacctacagcgtgagctatgagaaagcgccacgcttcccgaagggagaaaggcggacaggtatccggt
aagcggcagggtcggaacaggagagcgcacgagggagcttccagggggaaacgcctggtatctttatagtcctgt
cgggtttcgccacctctgacttgagcgtcgatttttgtgatgctcgtcaggggggcggagcctatggaaaaacgc
cagcaacgcggcctttttacggttcctggccttttgctggccttttgctcacatgttctttcctgcgttatcccc
tgattctgtggataaccgtattaccgcctttgagtgagctgataccgctcgccgcagccgaacgaccgagcgcag
cgagtcagtgagcgaggaagcggaagagcgcctgatgcggtattttctccttacgcatctgtgcggtatttcaca
ccgcatatatggtgcactctcagtacaatctgctctgatgccgcatagttaagccagtatacactccgctatcgc
tacgtgactgggtcatggctgcgccccgacacccgccaacacccgctgacgcgccctgacgggcttgtctgctcc
cggcatccgcttacagacaagctgtgaccgtctccgggagctgcatgtgtcagaggttttcaccgtcatcaccga
aacgcgcgaggcagctgcggtaaagctcatcagcgtggtcgtgaagcgattcacagatgtctgcctgttcatccg
cgtccagctcgttgagtttctccagaagcgttaatgtctggcttctgataaagcgggccatgttaagggcggttt
tttcctgtttggtcactgatgcctccgtgtaagggggatttctgttcatgggggtaatgataccgatgaaacgag
agaggatgctcacgatacgggttactgatgatgaacatgcccggttactggaacgttgtgagggtaaacaactgg
cggtatggatgcggcgggaccagagaaaaatcactcagggtcaatgccagcgcttcgttaatacagatgtaggtg
ttccacagggtagccagcagcatcctgcgatgcagatccggaacataatggtgcagggcgctgacttccgcgttt
ccagactttacgaaacacggaaaccgaagaccattcatgttgttgctcaggtcgcagacgttttgcagcagcagt
cgcttcacgttcgctcgcgtatcggtgattcattctgctaaccagtaaggcaaccccgccagcctagccgggtcc
tcaacgacaggagcacgatcatgcgcacccgtggccaggacccaacgctgcccgagatct

5.2.2 p15A-xR

Plasmid for the expression of OC6, C4, and OC12 receptors, LuxR, RhlR and LasR (xR). Their sequences are provided below.

J23106 :: RBS :: xR :: TT BBa_B0015 :: p15A ori :: KanR

aacaccccttgtattactgtttatgtaagcagacagttttattgttcatgatgatatatttttatcttgtgcaat
gtaacatcagagGAGTTACCTGCAGCGATTGTTTACGGCTAGCTCAGTCCTAGGTATAGTGCTAGCCTCATTCGC
TAATCGCCACGACGCGTAGTCGACGACCAGCTTTCTTAAGGGTACCAAGAAGGAGATATACATXXXXXXXXXXXX
XXXXXXXXXXXXXXXXXXXXXXXXXXXXXXXXXXXXXXXXXXXXXXXXXXXXXXXXXXXXXXXXXXXXXXXXXXX
XXXXXXXXXXXXXXXXXXXXXXXXXXXXXXXXXXXXXXXXXXXCCATGGGGCGCGCCTGCTGCCCAGGCATCAAA
TAAAACGAAAGGCTCAGTCGAAAGACTGGGCCTTTCGTTTTATCTGTTGTTTGTCGGTGAACGCTCTCTACTAGA
GTCACACTGGCTCACCTTCGGGTGGGCCTTTCTGCGTTTATATCAATTGGAATTCagaaaaaaggctgcaccggt
gcgtcagcagaatatgtgatacaggatatattccgcttcctcgctcactgactcgctacgctcggtcgttcgact
gcggcgagcggaaatggcttacgaacggggcggagatttcctggaagatgccaggaagatacttaacagggaagt
gagagggccgcggcaaagccgtttttccataggctccgcccccctgacaagcatcacgaaatctgacgctcaaat
cagtggtggcgaaacccgacaggactataaagataccaggcgtttccccctggcggctccctcgtgcgctctcct
gttcctgcctttcggtttaccggtgtcattccgctgttatggccgcgtttgtctcattccacgcctgacactcag
ttccgggtaggcagttcgctccaagctggactgtatgcacgaaccccccgttcagtccgaccgctgcgccttatc
cggtaactatcgtcttgagtccaacccggaaagacatgcaaaagcaccactggcagcagccactggtaattgatt
tagaggagttagtcttgaagtcatgcgccggttaaggctaaactgaaaggacaagttttggtgactgcgctcctc
caagccagttacctcggttcaaagagttggtagctcagagaaccttcgaaaaaccgccctgcaaggcggtttttt
cgttttcagagcaagagattacgcgcagaccaaaacgatctcaagaagatcatcttattaaggggtctgacgctc
agtggaacgaaaactcacgttaagggattttggtcatgagattatcaaaaaggatcttcacctagatcctttta
tgagtaaacttggtctgacagttaccaatgcttaatcagtgaggcacctatctcagcgatctgtctatttcgttc
atccatagttgcctgactccccgtcgtgtagataactacgatacgggagggcttaccatctggccccagtgctgc
aatgataccgcgagacccacgctcaccggctccagatttatcagcaataaaccagccagccgattcgagctcgcc
ccggggatcgaccagttggtgattttgaacttttgctttgccacggaacggtctgcgttgtcgggaagatgcgtg
atctgatccttcaactcagcaaaagttcgatttattcaacaaagccgccgtcccgtcaagtcagcgtaatgctct
gccagtgttacaaccaattaaccaattctgattagaaaaactcatcgagcatcaaatgaaactgcaatttattca
tatcaggattatcaataccatatttttgaaaaagccgtttctgtaatgaaggagaaaactcaccgaggcagttcc
ataggatggcaagatcctggtatcggtctgcgattccgactcgtccaacatcaatacaacctattaatttcccct
cgtcaaaaataaggttatcaagtgagaaatcaccatgagtgacgactgaatccggtgagaatggcaaaagcttat
gcatttctttccagacttgttcaacaggccagccattacgctcgtcatcaaaatcactcgcatcaaccaaaccgt
tattcattcgtgattgcgcctgagcgagacgaaatacgcgatcgctgttaaaaggacaattacaaacaggaatcg
aatgcaaccggcgcaggaacactgccagcgcatcaacaatattttcacctgaatcaggatattcttctaatacct
ggaatgctgttttcccggggatcgcagtggtgagtaaccatgcatcatcaggagtacggataaaatgcttgatgg
tcggaagaggcataaattccgtcagccagtttagtctgaccatctcatctgtaacatcattggcaacgctacctt
tgccatgtttcagaaacaactctggcgcatcgggcttcccatacaatcgatagattgtcgcacctgattgcccga
cattatcgcgagcccatttatacccatataaatcagcatccatgttggaatttaatcgcggcctcgagcaagacg
tttcccgttgaatatggctcat

5.2.3 pCC1R

Receptor array including the pC and OHC14 receptors RpaR and CinR. The AiiA lactonase gene is also present under an inducible PphlF promoter; this is not used in this study.

TT L3S3P21 :: BBa_J23101 :: RBS pca3 :: pcaU^{AM} :: RBS nah3 :: nahR^{AM} :: RBS BBa_B0034 :: rpaR :: TT BBa_B1006 :: BBa_J23119 :: RBS :: phlF^{AM} :: RBS :: cinR^{AM} :: RBS :: vanR^{AM} :: TT L3S2P21 :: PphlF :: riboJ :: RBS BBa_B0064 :: aiiA :: TT IOT :: camR :: repE ori

CCAATTATTGAAGGCCTCCCTAACGGGGGGCCTTTTTTTGTTTCTGGTCTCCCGCTTAACGATCGTTGGCTGGAG
ATTTTGAGGGTCGAATtttacagctagctcagtcctaggtattatgctagctcatgaCGCTTACAATAGACGAAC
AATAAAGGAGGAATTAACCGATGTGGTCGAACATGGATGACAAGAAAGTGAAAGAGGAGAATATTCTGCACAATT
CCACCAACAAGAAGATCATCCGCCACGAAGATTTTGTAGCCGGCATTAGCAAAGGGATGGCGATTCTGGATTCGT
TTGGTACAGATCGTCATCGCCTCAATATCACCATGGCCGCAGAGAAAACCGGTATGACACGTGCAGCAGCTCGTC
GCCACCTGCTTACTCTGGAGTATCTGGGCTATCTGGAAAGTGACGGCCACTACTTCTACTTAACTCCCAAAATCC
TGAAATTCAGTGGTTCATATTTGGGTGGTGCTCAATTGCCGAAAATTTCCCAACCACTGTTGAACTTGCTTACGA
CCCAGACCAGCCTGATTTACAGCGTGATGGTGTTGGATGGCTATGAAGCCATTACCATTGCGCGTTCTGCCGCTC
ATCAGCAAACCGACCGCGTTAACCCGTATGGTTTACATCTCGGGAATCGCTTACCAGCGCATACAACGTCAGCGG
GCAAAATCCTGTTAGCGTATTTGGATGACCATGCCCAGCAAGAGTGGCTCAATCAGTACCCTCTGCAACGGCTCA
CGAAATACACGTATACCAACCACATCGACTTTCTGCGCCTTTTGAGTGAAATCAAGGAACAGGGTTGGTGCTATA
GTTCGGAAGAACACGAACTGGGAGTACACGCCCTTGCGGTTCCGATTTACGGACAACAGTCTCGCGTCGTAGCGG
CACTGAACATTGTCAGCCCGACAATGCGGACCACGAAAGAATACCTGATTCAGCATATTCTGCCGTTACTGCAAG
AAACTGCGCGTGAATTGCGCAATATCCTGTAATGAACCCCCTATAAGAAAAAGACTTAACTATCCATGGAACTGC
GTGACCTTGATTTAAACCTGCTGGTGGTGTTCAACCAGTTGCTGGTCGACAGACGCGTCTCTGTCACTGCGGAGA
ACCTGGGCCTGACCCAGCCTGCCGTGAGCAATGCGCTGAAACGCCTGCGCACCTCGCTACAGGACCCACTCTTCG
TGCGCACACATCAGGGAATGGAACCCACACCCTATGCCGCGCATCTGGCCGAGCACGTCACTTCGGCCATGCACG
CACTGCGCAACGCCCTACAGCACCATGAAAGCTTCGATCCGCTGACCAGCGAGCGTACCTTCACCCTGGCCATGA
CCGACATTGGCGAGATCTACTTCATGCCGCGGCTGATGGATGCGCTGGCTCACCAGGCCCCCAATTGCGTGATCA
GTACGGTGCGCGACAGTTCGATGAGCCTGATGCAGGCCTTGCAGAACGGAACCGTGGACTTGGCCGTGGGCCTGC
TTCCCAATCTGCAAACTGGCTTCTTTCAGCGCCGGCTGCTCCGTAATCACTACGTGTGCCTATGTCGCAAGGACC
ATCCAGTCACCCGCGAACCCCTGACTCTGGAGCGCTTCTGTTCCTACGGCCACGTGCGTGTCATCGCCGCTGGCA
CCGGCCACGGCGAGGTGGACACGTACATGACACGGGTCGGCATCCGGCGCGACATCCGTCTGGAAGTGCCGCACT
TCGCCGCCGTTGGCCACATCCTCCAGCGCACCGATCTGCTCGCCACTGTGCCGATATGTTTAGCCGACTGCTGCG
TAGAGCCCTTCGGCCTAAGCGCCTTGCCGCACCCAGTCGTCTTGCCTGAAATAGCCATCAACATGTTCTGGCATG
CGAAGTACCACAAGGACCTAGCCAATATTTGGTTGCGGCAACTGATGTTTGACCTGTTTACGGATTGATAAGAAT
TAAAGAGGGGAAAGGTACCATGATCGTCGGCGAAGATCAGCTTTGGGGACGGCGTGCGCTGGAGTTCGTCGATTC
CGTCGAACGGCTCGAGGCGCCGGCGCTGATCAGCCGGTTCGAATCGCTGATCGCGAGCTGCGGATTTACCGCCTA
CATCATGGCCGGCCTGCCGTCGCGCAATGCCGGACTACCGGAGCTGACGCTGGCCAATGGCTGGCCGCGAGACTG
GTTCGATCTGTATGTCAGCGAAAACTTCAGCGCGGTCGATCCGGTGCCGCGCCACGGCGCTACCACGGTTCATCC
TTTCGTATGGTCCGATGCACCCTACGACCGCGACCGTGATCCGGCCGCCCACCGGGTCATGACCCGGGCGGCGGA
GTTCGGACTGGTCGAGGGTTACTGCATTCCGCTGCACTACGACGACGGTAGCGCCGCGATCAGCATGGCCGGCAA
AGATCCGGACCTCAGCCCGGCCGCGCGCGGCGCGATGCAGCTGGTCAGCATCTACGCGCATAGTCGCCTGCGCGC
ACTCAGCCGGCCAAAGCCGATCCGGCGCAACCGGCTCACGCCGCGCGAGTGCGAGATCCTGCAATGGGCAGCGCA
GGGCAAGACCGCCTGGGAAATCTCGGTAATCCTCTGCATCACCGAACGCACGGTGAAATTCCATCTGATCGAAGC
CGCCCGCAAGCTCGACGCCGCCAACCGCACCGCGGCGGTTGCCAAGGCATTGACGCTCGGATTGATCCGTTTGTG
AAATTCaaaaaaaaaccccgcccctgacagggcggggttttttttTGAGATTTTGAGACACAAGGTCGAAtCGCAC
CAAGACAGGTTTGTCCATTGACAGCTAGCTCAGTCCTAGGTATAATGCTAGCCTATGGACTATGTTTGAAAGGGA
GAAATACTAGATGGCACGTACCCCGAGCCGTAGCAGCATTGGTAGCCTGCGTAGTCCGCATACCCATAAAGCAAT
TCTGACCAGCACCATTGAAATCCTGAAAGAATGTGGTTATAGCGGTCTGAGCATTGAAAGCGTGGCACGTCGCGC
CGGTGCAGGCAAACCGACCATTTATCGTTGGTGGACCAACAAAGCAGCACTGATTGCCGAAGTGTATGAAAATGA
AATCGAACAGGTACGTAAATTTCCGGATTTGGGTAGCTTTAAAGCCGATCTGGATTTTCTGCTGCATAATCTGTG
GAAAGTTTGGCGTGAAACCATTTGTGGTGAAGCATTTCGTTGTGTTATTGCAGAAGCACAGTTGGACCCTGTAAC
CCTGACCCAACTGAAAGATCAGTTTATGGAACGTCGTCGTGAGATACCGAAAAAACTGGTTGAAGATGCCATTAG
CAATGGTGAACTGCCGAAAGATATCAATCGTGAACTGCTGCTGGATATGATTTTTGGTTTTTGTTGGTATCGCCT
GCTGACCGAACAGTTGACCGTTGAACAGGATATTGAAGAATTTACCTTCCTGCTGATTAATGGTGTTTGTCCGGG

TACACAGTGTTGATGAAGGTCCGAGACGCCCGTCAACGGAGAACGGCGAATGATTGAGAATACCTATAGCGAAAA
GTTCGAGTCCGCGTTCGAACAGATCAAAGCGGCGGCCAACGTGGATGCCGCCATCCGTATTCTCCAGGCGGAATA
TAACCTCGATTTCGTCACCTACCATCTCGCCCAGACAATCGCGAGCAAGATCGATTCGCCCTTCGTGCGCACCAC
CTATCCGGATGCCTGGGTTTCCCGTTACCTCCTCAACTGCTATGTGAAGGTCGATCCGATCATCAAGCAGGGCTT
CGAACGCCAGCTGCCCTTCGACTGGAGCGAGGTCGAACCGACGCCGGAGGCCTATGCCATGCTGGTCGACGCCCA
GAAACACGGCATCGATGACAATGGCTACTCCATCCCCGTCGCCGACAAGGCGCAGCGCCGCGCCCTGCTGTCGCT
GAATGCCCATATACCGGCCGACGAATGGACCGAGCTCGTGCGCCGCTGCCGCAATGAGTGGATCGAGATCGCCCA
TCTGATCCACCGCAAGGCCGTATATGAGCTGCATGGCGAAAACGATCCGGTGCCGGCATTGTCGCCGCGCGAGAT
CGAGTGTCTGCACTGGACCGCCCTCGGCAAGGATTACAAGGATATTTCGGTCATCCTGGGCATATCAGAGCATAC
CACACGCGATTACCTGAAAACCGCCCGCTTCAGGCTCGGCTGCACCACGATCTCGGCCGCCGCGTCGCGGGCTGT
TCAATTGCGCATCATCAATCCCTATAGGATCCGCATGACGCGACGTAATTGGTAATGAGCTTAAACTAACGAACG
TAAATAAGGAGGATAGACATGGACATGCCTCGTATTAAACCGGGTCAGCGTGTTATGATGGCACTGCGTAAAATG
ATTGCAAGCGGTGAAATCAAAAGTGGTGAACGTATTGCAGAAATTCCGACCGCAGCAGCACTGGGTGTTAGCCGT
ATGCCGGTTCGTATCGCACTGCGTTCACTGGAACAAGAAGGTCTGGTTGTTCGTCTGGGTGCACGTGGTTATGCA
GCCCGTGGTGTTAGCAGCGATCAGATTCGTGATGCAATTGAAGTTCGTGGTGTTCTGGAAGGTTTTGCAGCACGT
CGTCTGGCAGAACGTGGTATGACCGCAGAAACCCATGCACGTTTTGTTGTACTGATTGCAGAAGGTGAAGCACTG
TTTGCAGCCGGTCGCCTGAATGGTGAAGATCTGGATCGTTATGCCGCATATAATCAGGCATTTCATGATACCCTG
GTTAGCGCAGCAGGTAATGGTGCAGTTGAAAGCGCACTGGCACGTAATGGTTTTGAACCGTTTGCAGCAGCCGGT
GCACTGGCCCTGGATCTGATGGACCTGTCTGCCGAATATGAACATCTGCTGGCAGCACATCGTCAGCATCAGGCA
GTTCTGGATGCAGTTAGCTGTGGTGATGCCGAAGGTGCAGAACGTATTATGCGTGATCATGCACTGGCAGCAATT
CGTAATGCAAAAGTTTTTGAAGCAGCAGCAAGCGCAGGCGCACCGCTGGGTGCAGCATGGTCAATTCGTGCAGAT
TGATAACTCGGTACCAAATTCCAGAAAAGAGGCCTCCCGAAAGGGGGGCCTTTTTTCGTTTTGGTCCCGACGTAC
GGTGGAATCTGATTCGTTACCAATTGACATGATACGAAACGTACCGTATCGTTAAGGTAGCTGTCACCGGATGTG
CTTTCCGGTCTGATGAGTCCGTGAGGACGAAACAGCCTCTACAAATAATTTTGTTTAATCTAGAGAAAGAGGGGA
AATACTAGatgacagtaaagaagctttatttcgtcccagcaggtcgttgtatgttggatcattcgtctgttaata
gtacattaacaccaggagaattattagacttaccggtttggtgttatcttttggagactgaagaaggacctattt
tagtagatacaggtatgccagaaagtgcagttaataatgaaggtctttttaacggtacatttgtcgaagggcagg
ttttaccgaaaatgactgaagaagatagaatcgtgaatattttaaaacgggttggttatgagccggaagaccttc
tttatattattagttctcacttgcattttgatcatgcaggaggaaatggcgcttttataaatacaccaatcattg
tacagcgtgctgaatatgaggcggcgcagcatagcgaagaatatttgaaagaatgtatattgccgaatttaaact
acaaaatcattgaaggtgattatgaagtcgtaccaggagttcaattattgcatacaccaggccatactccagggc
atcaatcgctattaattgagacagaaaaatccggtcctgtattattaacgattgatgcatcgtatacgaaagaga
attttgaaaatgaagtgccatttgcgggatttgattcagaattagctttatcttcaattaaacgtttaaaagaag
tggtgatgaaagagaagccgattgttttctttggacatgatatagagcaggaaaggggatgtaaagtgttccctg
aatatatatgataaATAGTAATTGGTAACGAATCAGACAATTGACGGCTCGAGGGAGTAGCATAGGGTTTGCAGA
ATCCCTGCTTCGTCCATTTGACAGGCACATTATGCATCGATGATAAGCTGTCAAACATGAGCAGATCCTCTACGC
CGGACGCATCGTGGCCGGCATCACCGGCGCCACAGGTGCGGTTGCTGGCGCCTATATCGCCGACATCACCGATGG
GGAAGATCGGGCTCGCCACTTCGGGCTCATGAGCAAATATTTTATCTGTGACCAAATTCTCAGTGGTTGTCGCAG
GCGGTGGAAGCACCTTTACGCCActgcagggcgtaatcatggtcatagctgtttcctgtgtgaaattgttatccg
ctcacaattccacacaacatacgagccggaagcataaagtgtaaagcctggggtgcctaatgagtgagctaactc
acattaattgcgttgcgctcactgcccgctttccagtcgggaaacctgtcgtgccagctgcattaatgaatcggc
caacgcgaaccccttgcggccgcccgggccgtcgaccaattctcatgtttgacagcttatcatcgaatttctgcc
attcatccgcttattatcacttattcaggcgtagcaaccaggcgtttaagggcaccaataactgccttaaaaaaa
ttacgccccgccctgccactcatcgcagtactgttgtaattcattaagcattctgccgacatggaagccatcaca
aacggcatgatgaacctgaatcgccagcggcatcagcaccttgtcgccttgcgtataatatttgcccatggtgaa
aacgggggcgaagaagttgtccatattggccacgtttaaatcaaaactggtgaaactcacccagggattggctga
gacgaaaaacatattctcaataaaccctttagggaaataggccaggttttcaccgtaacacgccacatcttgcga
atatatgtgtagaaactgccggaaatcgtcgtggtattcactccagagcgatgaaaacgtttcagtttgctcatg
gaaaacggtgtaacaagggtgaacactatcccatatcaccagctcaccgtctttcattgccatacgaattccgg
atgagcattcatcaggcgggcaagaatgtgaataaaggccggataaaacttgtgcttatttttctttacggtctt
taaaaaggccgtaatatccagctgaacggtctggttataggtacattgagcaactgactgaaatgcctcaaaatg
ttctttacgatgccattgggatatatcaacggtggtatatccagtgatttttttctccattttagcttccttagc
tcctgaaaatctcgataactcaaaaaatacgcccggtagtgatcttatttcattatggtgaaagttggaacctct
tacgtgccgatcaacgtctcattttcgccaaaagttggcccagggcttcccggtatcaacagggacaccaggatt
tatttattctgcgaagtgatcttccgtcacaggtatttattcgcgataagctcatggagcggcgtaaccgtcgca
caggaaggacagagaaagcgcggatctgggaagtgacggacagaacggtcaggacctggattggggaggcggttg
ccgccgctgctgctgacggtgtgacgttctctgttccggtcacaccacatacgttccgccattcctatgcgatgc
acatgctgtatgccggtataccgctgaaagttctgcaaagcctgatgggacataagtccatcagttcaacggaag
tctacacgaaggttttgcgctggatgtggctgcccggcaccgggtgcagtttgcgatgccggagtctgatgcgg
ttgcgatgctgaaacaattatcctgagaataaatgccttggcctttatatggaaatgtggaactgagtggatatg
ctgtttttgtctgttaaacagagaagctggctgttatccactgagaagcgaacgaaacagtcgggaaaatctccc

attatcgtagagatccgcattattaatctcaggagcctgtgtagcgtttataggaagtagtgttctgtcatgatg
cctgcaagcggtaacgaaaacgatttgaatatgccttcaggaacaatagaaatcttcgtgcggtgttacgttgaa
gtggagcggattatgtcagcaatggacagaacaacctaatgaacacagaaccatgatgtggtctgtccttttaca
gccagtagtgctcgccgcagtcgagcgacagggcgaagccctcggctggttgccctcgccgctgggctggcggcc
gtctatggccctgcaaacgcgccagaaacgccgtcgaagccgtgtgcgagacaccgcggccggccgccggcgttg
tggatacctcgcggaaaacttggccctcactgacagatgaggggcggacgttgacacttgaggggccgactcacc
cggcgcggcgttgacagatgaggggcaggctcgatttcggccggcgacgtggagctggccagcctcgcaaatcgg
cgaaaacgcctgattttacgcgagtttcccacagatgatgtggacaagcctggggataagtgccctgcggtattg
acacttgaggggcgcgactactgacagatgaggggcgcgatccttgacacttgaggggcagagtgctgacagatg
aggggcgcacctattgacatttgaggggctgtccacaggcagaaaatccagcatttgcaagggtttccgcccgtt
tttcggccaccgctaacctgtcttttaacctgcttttaaaccaatatttataaaccttgtttttaaccagggctg
cgccctgtgcgcgtgaccgcgcacgccgaaggggggtgccccccttctcgaaccctcccggtcgagtgagcgag
gaagcaccagggaacagcacttatatattctgcttacacacgatgcctgaaaaaacttcccttggggttatccac
ttatccacggggatatttttataattattttttttatagtttttagatcttctttttttagagcgccttgtaggcc
tttatccatgctggttctagagaaggtgttgtgacaaattgccctttcagtgtgacaaatcaccctcaaatgaca
gtcctgtctgtgacaaattgcccttaaccctgtgacaaattgccctcagaagaagctgtttttttcacaaagttat
ccctgcttattgactctttttttatttagtgtgacaatctaaaaacttgtcacacttcacatggatctgtcatggc
ggaaacagcggttatcaatcacaagaaacgtaaaaatagcccgcgaatcgtccagtcaaacgacctcactgaggc
ggcatatagtctctcccgggatcaaaaacgtatgctgtatctgttcgttgaccagatcagaaaatctgatggcac
cctacaggaacatgacggtatctgcgagatccatgttgctaaatatgctgaaatattcggattgacctctgcgga
agccagtaaggatatacggcaggcattgaagagtttcgcggggaaggaagtggtttttttatcgccctgaagagga
tgccggcgatgaaaaaggctatgaatcttttccttggtttatcaaacgtgcgcacagtccatccagagggcttta
cagtgtacatatcaacccatatctcattcccttctttatcgggttacagaaccggtttacgcagtttcggcttag
tgaaacaaaagaaatcaccaatccgtatgccatgcgtttatacgaatccctgtgtcagtatcgtaagccggatgg
ctcaggcatcgtctctctgaaaatcgactggatcatagagcgttaccagctgcctcaaagttaccagcgtatgcc
tgacttccgccgccgcttcctgcaggtctgtgttaatgagatcaacagcagaactccaatgcgcctctcatacat
tgagaaaaagaaaggccgccagacgactcatatcgtattttccttccgcgatatcacttccatgacgacaggata
gtctgagggttatctgtcacagatttgagggtggttcgtcacatttgttctgacctactgagggtaatttgtcac
agttttgctgtttccttcagcctgcatggatttctcatactttttgaactgtaattttaaggaagccaaattt
gagggcagtttgtcacagttgatttccttctctttcccttcgtcatgtgacctgatatcggggttagttcgtca
tcattgatgagggttgattatcacagtttattactctgaattggctatccgcgtgtgtacctctacctggagttt
ttcccacggtggatatttcttcttgcgctgagcgtaagagctatctgacagaacagttcttctttgcttcctcgc
cagttcgctcgctatgctcggttacacggctgcggcggatgtgctgcaaggcgattaagttgggtaacgccaggg
ttttcccagtcacgacgttgtaaaacgacggccagtgcggccgcTAACCAATCAGGCTTCCTACTTACAGAATTG
AGAAAAGAGGATGTGGAA

6 References

(1) Karig, D.; Martini, K. M.; Lu, T.; DeLateur, N. A.; Goldenfeld, N.; Weiss, R.; Michael Martini, K.; Lu, T.; DeLateur, N. A.; Goldenfeld, N.; Weiss, R. Stochastic Turing Patterns in a Synthetic Bacterial Population. *Proc Natl Acad Sci U S A* **2018**, *115* (26), 6572–6577. https://doi.org/10.1073/pnas.1720770115.

(2) Friedman, L. Structure of Agar Gels from Studies of Diffusion. *J Am Chem Soc* **1930**, *52* (4), 1311–1314. https://doi.org/10.1021/ja01367a003.

(3) Savka, M. A.; Le, P. T.; Burr, T. J. LasR Receptor for Detection of Long-Chain Quorum-Sensing Signals: Identification of N-Acyl-Homoserine Lactones Encoded by the AvsI Locus of Agrobacterium Vitis. *Curr Microbiol* **2011**, *62* (1), 101–110. https://doi.org/10.1007/s00284-010-9679-1.

(4) Du, P.; Zhao, H.; Zhang, H.; Wang, R.; Huang, J.; Tian, Y.; Luo, X.; Luo, X.; Wang, M.; Xiang, Y.; Qian, L.; Chen, Y.; Tao, Y.; Lou, C. De Novo Design of an Intercellular Signaling Toolbox for Multi-Channel Cell–Cell Communication and Biological Computation. *Nat Commun* **2020**, *11* (1), 4226. https://doi.org/10.1038/s41467-020-17993-w.

(5) Zucca, S.; Pasotti, L.; Politi, N.; Casanova, M.; Mazzini, G.; Cusella De Angelis, M. G.; Magni, P. Multi-Faceted Characterization of a Novel LuxR-Repressible Promoter Library for Escherichia Coli. *PLoS One* **2015**, *10* (5). https://doi.org/10.1371/JOURNAL.PONE.0126264.

(6) Meyer, A. J.; Segall-Shapiro, T. H.; Glassey, E.; Zhang, J.; Voigt, C. A. Escherichia Coli "Marionette" Strains with 12 Highly Optimized Small-Molecule Sensors. *Nat Chem Biol* **2019**, *15* (2), 196–204. https://doi.org/10.1038/s41589-018-0168-3.

(7) Balleza, E.; Kim, J. M.; Cluzel, P. Systematic Characterization of Maturation Time of Fluorescent Proteins in Living Cells. *Nature Methods 2017 15:1* **2017**, *15* (1), 47–51. https://doi.org/10.1038/nmeth.4509.
